# Transcription of a 5’ extended mRNA isoform directs dynamic chromatin changes and interference of a downstream promoter

**DOI:** 10.7554/eLife.27420

**Published:** 2017-09-14

**Authors:** Minghao Chia, Amy Tresenrider, Jingxun Chen, Gianpiero Spedale, Victoria Jorgensen, Elçin Ünal, Folkert Jacobus van Werven

**Affiliations:** 1 The Francis Crick InstituteLondonUnited Kingdom; 2 Department of Molecular and Cell BiologyUniversity of California, BerkeleyBerkeleyUnited States; Howard Hughes Medical Institute, Memorial Sloan Kettering Cancer CenterUnited States

**Keywords:** transcription, chromatin, meiosis, interference, mRNA isoform, promoter, *S. cerevisiae*

## Abstract

Cell differentiation programs require dynamic regulation of gene expression. During meiotic prophase in *Saccharomyces cerevisiae*, expression of the kinetochore complex subunit Ndc80 is downregulated by a 5’ extended long undecoded *NDC80* transcript isoform. Here we demonstrate a transcriptional interference mechanism that is responsible for inhibiting expression of the coding *NDC80* mRNA isoform. Transcription from a distal *NDC80* promoter directs Set1-dependent histone H3K4 dimethylation and Set2-dependent H3K36 trimethylation to establish a repressive chromatin state in the downstream canonical *NDC80* promoter. As a consequence, *NDC80* expression is repressed during meiotic prophase. The transcriptional mechanism described here is rapidly reversible, adaptable to fine-tune gene expression, and relies on Set2 and the Set3 histone deacetylase complex. Thus, expression of a 5’ extended mRNA isoform causes transcriptional interference at the downstream promoter. We demonstrate that this is an effective mechanism to promote dynamic changes in gene expression during cell differentiation.

## Introduction

Cell fate programs are driven by underlying gene regulatory networks. The budding yeast gametogenesis program, also known as sporulation, provides an ideal model for understanding principles of cell fate progression. In sporulation a diploid cell exits the mitotic program and produces four haploid gametes packaged as spores. Gametogenesis is driven by a specialized cell division called meiosis (Marston and Amon, 2004). After entering meiosis, diploid cells undergo a single round of DNA replication, and then recombination of homologous chromosomes in a long-lasting prophase. Completion of meiotic prophase is followed by two rounds of chromosome segregation as well as development and packaging of meiotic gametes into spores. In the budding yeast *S. cerevisiae*, meiosis can be induced synchronously, allowing the study of stage-specific regulation of gene expression.

The yeast meiotic program is dynamically regulated by sequential waves of gene expression (Chu et al., 1998; Primig et al., 2000). Two master transcription factors, Ime1 and Ndt80, control gene expression during meiosis (Kassir et al., 1988; Xu et al., 1995). Ime1 regulates the early genes controlling S phase and prophase, whereas Ndt80 induces sets of genes controlling meiotic chromosome segregation and spore formation (Mitchell et al., 1990; Chu and Herskowitz, 1998). These two transcription factors are critical for stage specific gene expression during meiosis.

Noncoding RNAs (ncRNAs) and alternate mRNA isoforms are expressed throughout yeast meiosis (Brar et al., 2012; Kim Guisbert et al., 2012; Lardenois et al., 2015). In addition, ncRNAs are transcribed from the 3’ end of genes important for meiosis and sporulation (Zhang et al., 2011). However, only a limited number of these ncRNAs have been assigned a biological function. For example, in cells with a single mating type locus (*MAT*a or *MAT*α) transcription of ncRNAs represses *IME1* and *IME4*, two regulators of entry in meiosis (Hongay et al., 2006; van Werven et al., 2012). Throughout meiosis a subset of genes show stage specific expression of mRNA isoforms with often reduced translational capabilities (Brar et al., 2012). However, it is not well understood how ncRNAs and mRNA isoforms contribute to dynamic control of gene expression during yeast meiosis.

One gene with a dynamic expression pattern during yeast meiosis encodes for the kinetochore subunit Ndc80. Ndc80 is downregulated during meiotic prophase and is rapidly induced during meiotic chromosome segregation (Miller et al., 2012; Meyer et al., 2015). In the presence of a spindle, mis-expression of Ndc80 during meiotic prophase causes aberrant meiotic chromosome segregation (Miller et al., 2012). Thus, the dynamic control of Ndc80 expression is critical for meiotic divisions. How Ndc80 is repressed during the early stages of meiosis remained elusive. In the accompanying paper, we show that the transcription of a 5’ extended *NDC80* long undecoded transcript isoform (*NDC80^luti^*) is responsible for repressing the coding-competent *NDC80* isoform (*NDC80^ORF^*) during meiotic S-phase and prophase (Chen et al., 2017). Furthermore, *NDC80^luti^* cannot be translated into Ndc80 protein due to translation of the upstream open reading frames in this mRNA isoform. Altogether, these results demonstrate that *NDC80^luti^* functions in a regulatory manner, in which its transcription is both necessary and sufficient to downregulate *NDC80^ORF^* levels during meiotic prophase.

In this work we describe the mechanism by which the 5’ extended *NDC80^luti^* mRNA isoform represses *NDC80^ORF^. NDC80^luti^* transcription interferes with the downstream *NDC80^ORF^* promoter by establishing a repressive chromatin state. This repression requires both Set3 and Set2. *NDC80^luti^*-mediated repression can be rapidly reversed to suit the physiological needs of the cell. Furthermore, the repression mechanism described here can be adapted to fine-tune gene expression. Thus, transcription of a 5’ extended mRNA isoform mediates transcriptional interference of the downstream promoter, allowing dynamic control of gene expression.

## Results

### Transcription of a *NDC80* mRNA isoform with an extended 5’ leader represses the canonical *NDC80* mRNA

**Figure 1.**
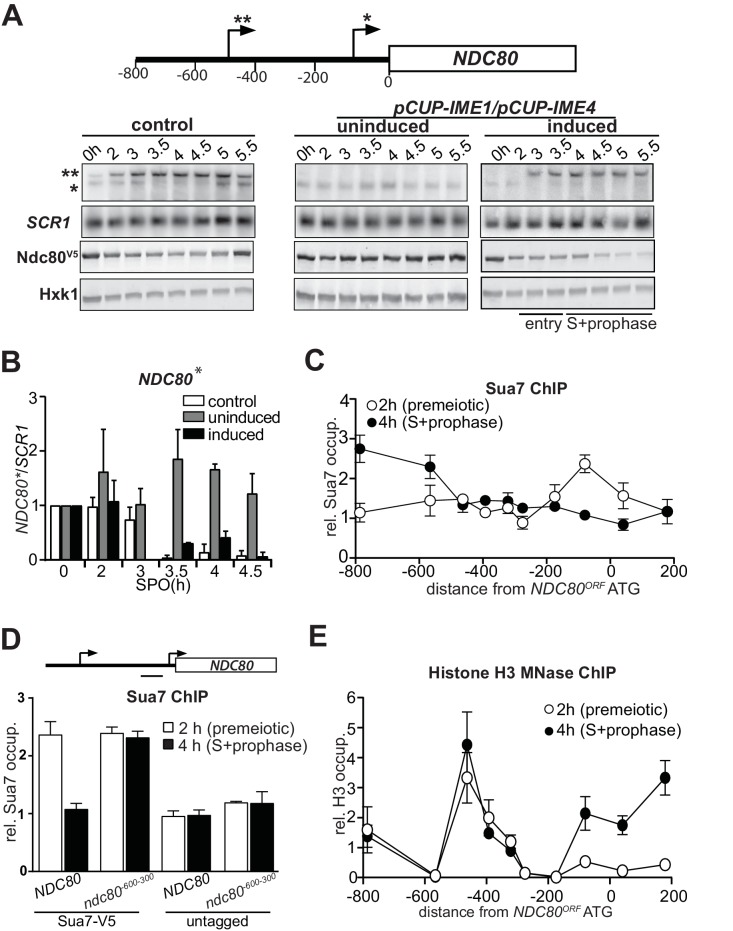
*NDC80^luti^* transcription represses the *NDC80^ORF^* promoter. (**A**) Expression pattern of two *NDC80* mRNA isoforms during starvation and early meiosis. Diploid control cells (FW4644) or cells harboring the *CUP1* promoter fused with *IME1* and *IME4* (*pCUP-IME1*/*pCUP-IME4*) (FW1902) were grown overnight in rich medium, shifted to pre-sporulation medium, and subsequently transferred to sporulation medium (SPO). These cells also harbored *NDC80* tagged at the carboxy-terminus with three copies of the V5 epitope. After two hours in SPO, *IME1* and *IME4* were induced with CuSO_4_ (50 μM) to set in motion synchronous meiosis in *pCUP-IME1*/*pCUP-IME4* cells. Samples were taken at the indicated time points for northern and western blot analyses. To detect the two different *NDC80* mRNA isoforms, RNA was extracted, separated by gel electrophoresis, blotted, and hybridized with a probe that spans the *NDC80* promoter and the 5’ end of the coding region. As a loading control, we also hybridized the blot with a probe specific for *SCR1*. Ndc80 protein levels were determined by western blot using anti V5 antibodies. As a loading control, we also detected Hxk1 levels with anti-Hxk1 antibodies. A schematic of the *NDC80* locus is shown over the northern blot. The single asterisk denotes the transcription start site of the short mitotic *NDC80* mRNA isoform. The double asterisk denotes the transcription start site of the distal 5’ extended long *NDC80* mRNA isoform. The distance in base pairs from the *NDC80* start codon is also displayed. (**B**) Quantification of expression of the short mitotic *NDC80* mRNA isoform from the proximal transcription start-site (labelled with one asterisk) up till the 4.5 hr time point. The mean of two independent repeats plus the standard error of the mean are displayed. The signal was normalized over *SCR1*. To control for technical variation between experiments and blots, the 0 hr time point was set to one. (**C**) *NDC80^luti^* transcription correlates with reduced TFIIB (Sua7) binding at the *NDC80^ORF^* promoter. The two *NDC80* isoforms described in A were defined as long undecoded transcript isoform (*NDC80^luti^*) and Ndc80 coding mRNA isoform (*NDC80^ORF^*), respectively. Cells harboring Sua7 tagged with three copies of V5 (Sua7-V5) and the *pCUP-IME1*/*pCUP-IME4* alleles (FW2957) were induced to undergo meiosis synchronously as described in A. Samples for chromatin immunoprecipitation were taken at two hours (2 hr (premeiotic), no *NDC80^luti^* transcription) and four hours after transfer to sporulation medium (SPO) (4 hr (S + prophase), *NDC80^luti^* transcription). Cells were fixed with formaldehyde, chromatin extracts were prepared and Sua7-V5 bound DNA fragments were immunoprecipitated using agarose beads coupled with anti-V5 antibodies. The recovered DNA fragments were quantified by qPCR using ten different primer pairs scanning the *NDC80* locus, and were normalized over a primer pair directed against the *HMR* locus. The midpoint position of each primer pair is indicated in the x-axis. The mean normalized signal from three independent experiments plus the standard error of the mean for each primer pair is displayed. ‘rel.’ means relative; ‘occup.”, occupancy. (**D**) *NDC80^luti^* transcription is required for inhibiting Sua7 binding in the *NDC80^ORF^* promoter during meiotic prophase. Similar analysis as C except that a mutant strain harboring a deletion upstream in the *NDC80* promoter region (*ndc80^-600-300^*, FW5530) and untagged strains (FW1902 and FW1868) were included in the analysis. A primer pair directed against a *NDC80^ORF^* core promoter was used for the quantification of Sua7 binding at the *NDC80^ORF^* promoter. (**E**) *NDC80^luti^* transcription correlates with the establishment of repressive chromatin in the promoter of *NDC80^ORF^.* Chromatin structure at the *NDC80* locus was determined by ChIP of histone H3 on micrococcal nuclease (MNase) treated extracts in cells that also harbored *pCUP-IME1/pCUP-IME4* (FW1902). Samples were taken at two hours (2 hr (premeiotic), no *NDC80^luti^* transcription) and four hours after transfer to SPO (4 hr (S + prophase), *NDC80^luti^* transcription), fixed with formaldehyde, and chromatin extracts were treated with micrococcal nuclease. Extracts that predominantly contained mononucleosomes were used for ChIP assays with histone H3 antibodies (see Materials and methods for details). The recovered DNA fragments were quantified by qPCR using ten different primer pairs directed against the *NDC80* locus relative to a no MNase input. The signals from each primer pair were then normalized over a primer pair directed against the *PHO5* core promoter. The midpoint position of each primer pair is indicated in the x-axis. The mean signal from three independent experiments plus the standard error of the mean for each primer pair is displayed.

Genome wide transcriptome data indicated that there are at least two different mRNA isoforms expressed from the *NDC80* locus (Brar et al., 2012; Kim Guisbert et al., 2012; Lardenois et al., 2015). During mitotic growth, an *NDC80* mRNA is produced from its core promoter. However, in early meiosis a longer *NDC80* mRNA is transcribed from approximately 500 base pairs (bp) upstream of the *NDC80* start codon. To investigate the expression pattern of the *NDC80* mRNA isoforms more closely in cells undergoing meiosis, we performed northern blot analyses using a probe that recognizes both the ORF and upstream region of *NDC80*. Cells were first grown in rich medium overnight and then shifted to pre-sporulation medium. After a second overnight growth, cells were transferred to sporulation medium (SPO) and samples were taken at specific intervals throughout meiosis. In wild-type cells entering meiosis, both *NDC80* mRNA isoforms were co-expressed (Figure 1A, left panel [control]). After 3 hr in SPO, the expression of the short mitotic isoform decreased, whereas the levels of the longer *NDC80* mRNA isoform increased. At 5 hr the short *NDC80* form was induced (Figure 1A, left panel [control]), which corresponds to cells entering meiotic divisions (Chen et al., 2017). To control for changes in RNA levels, the expression of unrelated RNA polymerase II and III genes, *CIT1* and *SCR1* respectively, was measured (Figure 1—figure supplement 1A and B). We observed that *CIT1* levels fluctuated throughout the meiotic time course, while *SCR1* expression was more constant (Figure 1A and Figure 1—figure supplement 1A and B). Hence, the expression levels of *NDC80* isoforms were normalized to *SCR1* (Figure 1B and Figure 1—figure supplement 1C). We also measured *NDC80* expression in a strain in which the *IME1* and *IME4* genes were fused to a copper inducible promoter (*pCUP-IME1* and *pCUP-IME4*). The induction of *pCUP-IME1* and *pCUP-IME4* ensures that cells can enter meiosis synchronously (Berchowitz et al., 2013; Chia and van Werven, 2016). Only the short mitotic isoform was clearly detected in cells arrested before entry into meiosis (Figure 1A, right panel (induced), and Figure 1B). Strikingly, soon after *IME1* and *IME4* were induced at two hours in SPO, expression of the long *NDC80* isoform increased and levels of the mitotic *NDC80* isoform decreased (Figure 1A, right panel (induced), and Figure 1B and Figure 1—figure supplement 1C). The mitotic *NDC80* isoform was repressed throughout meiotic S-and prophase. Mirroring changes in the levels of the mitotic *NDC80* transcript, Ndc80 protein levels also decreased during entry into meiosis, meiotic S-phase and prophase (Figure 1A and Figure 1—figure supplement 1D). As expected, expression of the mitotic *NDC80* isoform and Ndc80 protein remained relatively constant when meiosis was not induced (Figure 1A, middle panel (uninduced), Figure 1B and Figure 1—figure supplement 1D). We conclude that during meiotic entry, meiotic S phase and prophase, the expression of a longer *NDC80* form is induced and the mitotic form of *NDC80* is repressed.

**Figure 1—figure supplement 1.**
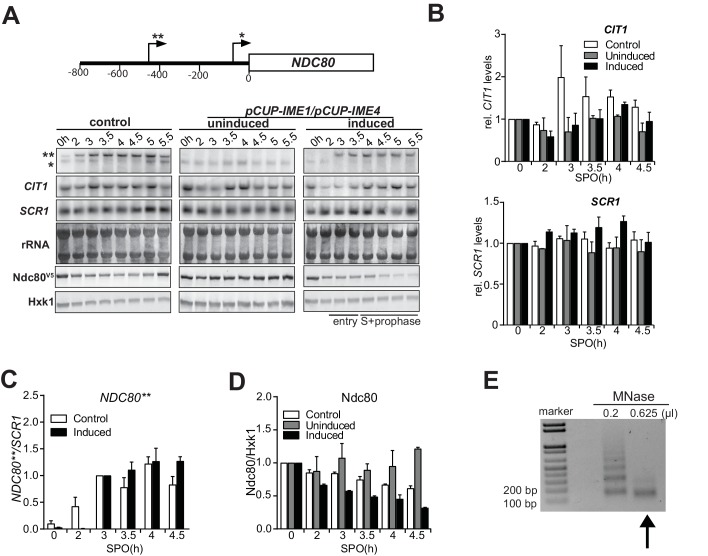
*NDC80^luti^* transcription represses the *NDC80^ORF^* promoter. (**A**) The same northern blot and western blot of *NDC80* expression during a meiotic time course as described in Figure 1A, except that *CIT1* and ribosomal RNA expression levels are also displayed. (**B**) Quantification of *CIT1* and *SCR1* northern blot signals from A up till the 4.5 hr time point. The mean of two independent repeats plus the standard error of the mean are displayed. (**C**) Quantification of expression of the long mRNA *NDC80* isoform described in Figure 1A (labelled with two asterisks). The mean of two independent repeats plus the standard error of the mean are displayed. The signal was normalized over *SCR1*. Since the long isoform is not expressed in premeiotic conditions, the 3 hr time point (as opposed to 0 hr) was set to one to control for technical variation between experiments and blots. (**D**) Quantification of Ndc80 protein levels as described in Figure 1A. The mean of two independent repeats plus the standard error of the mean are displayed. The signal was normalized over Hxk1. To control for technical variation between experiments and blots, the 0 hr time point was set to one. (**E**) Example of extract with mononucleosomes prepared from cells (FW1902, 2 hr [premeiotic]). In short, cells were fixed with formaldehyde, treated with zymolase, and subsequently treated with different concentrations of micrococcal nuclease (MNase). To check for the extent of MNase digestion, part of the sample from each extract was reverse crosslinked, purified, and separated by gel electrophoresis. The arrow indicates the extract that was used for subsequent ChIP analysis.

Our observation that the expression of the mitotic and longer *NDC80* mRNA isoforms are inversely correlated during early meiosis, suggests that there may be a direct effect of the longer *NDC80* isoform on mitotic *NDC80* repression. The accompanying paper by *Chen et al.* showed by a series of experiments that expression of the longer *NDC80* mRNA isoform is responsible for the decline in mitotic *NDC80* levels during early meiosis (Chen et al., 2017). Furthermore, *Chen et al.* showed that nine short upstream open reading frames in the extended 5’ region of the long isoform inhibit translation of Ndc80 protein from this mRNA isoform (Chen et al., 2017). Thus, the long *NDC80* mRNA isoform is translationally inert. Hence, this transcript has been defined as the *NDC80* long undecoded transcript isoform (*NDC80^luti^*). The short *NDC80* protein coding mRNA isoform is called *NDC80^ORF^*. This nomenclature is used thereafter.

### Transcription of *NDC80^luti^* correlates with reduced binding of TFIIB and repressive chromatin in the *NDC80^ORF^* promoter

The mechanism by which *NDC80^luti^* represses the downstream *NDC80^ORF^* promoter might be related to a transcriptional interference mechanism during which intergenic transcription or transcription over promoter regions establishes a repressive chromatin state and prevents transcription factors from binding (Martens et al., 2004; Hainer et al., 2011; van Werven et al., 2012). Similar to transcriptional interference, *NDC80^luti^*-mediated repression of *NDC80^ORF^* is exclusively *cis*-dominant (Martens et al., 2004; van Werven et al., 2012; Chen et al., 2017). To further investigate whether the mechanism of *NDC80^luti^*-mediated gene repression also shares other features of transcriptional interference, we tested whether *NDC80^luti^* transcription alters the association of transcription factors with the *NDC80^ORF^* promoter. The binding of the basal transcription factor Sua7 (TFIIB), which is homologous to human TFIIB, changed during meiosis across the *NDC80* locus (Figure 1C). Before entry into meiosis, Sua7 was bound to the core promoter of *NDC80^ORF^*. However, after *IME1* and *IME4* induction (four hours in SPO) when *NDC80^luti^* transcription occurred and cells underwent meiotic S phase, Sua7 binding to the *NDC80^ORF^* core promoter (around −100 bp from AUG) was reduced while binding to the *NDC80^luti^* promoter (around −600 bp from AUG) increased (Figure 1C). It is worth noting, that the signal for Sua7 binding also showed a peak at −800 bp, which may be due to fluctuation in expression of the adjacent *PAN6* gene in the divergent direction. Next, we examined Sua7 binding at the *NDC80^ORF^* promoter in a mutant that does not transcribe *NDC80^luti^* (*ndc80^-600-300^*) (Chen et al., 2017). In the *ndc80^-600-300^* mutant, no change in Sua7 binding around the *NDC80^ORF^* promoter was observed after induction of *IME1* and *IME4* (Figure 1D). This result shows that *NDC80^luti^* prevents TFIIB recruitment at the *NDC80^ORF^* promoter during early meiosis.

The reduction in TFIIB recruitment to the *NDC80^ORF^* promoter could be due to the establishment of a repressive chromatin state. For example, transcription of an intergenic ncRNA across the *SER3* promoter directs nucleosome assembly in the promoter, which is essential for *SER3* repression in budding yeast (Hainer et al., 2011). Therefore, we examined how the chromatin structure in the *NDC80^ORF^* promoter is modified by *NDC80^luti^* transcription. To identify where the nucleosomes stably associate with the *NDC80* locus, we performed chromatin immunoprecipitation (ChIP) of histone H3 on micrococcal nuclease (MNase) treated chromatin extracts (see material and methods for details) (Figure 1E and Figure 1—figure supplement 1E). In premeiotic cells (labeled ‘2 hr’ in Figure 1E) we detected a relatively low signal around the core promoter of *NDC80^ORF^*, which is indicative of a nucleosome free region (NFR) and consistent with active *NDC80^ORF^* transcription. During meiotic prophase (labeled ‘4 hr’ in Figure 1E), when *NDC80^luti^* was transcribed, the signal around the core promoter increased, indicating that nucleosome occupancy was increased. These findings are consistent with the notion that transcription of *NDC80^luti^* inhibits TFIIB recruitment and establishes a repressive chromatin state at the *NDC80^ORF^* promoter.

### *NDC80^luti^* transcription promotes Set1-dependent histone H3 lysine 4 dimethylation and Set2-dependent lysine 36 trimethylation in the *NDC80^ORF^* promoter

**Figure 2.**
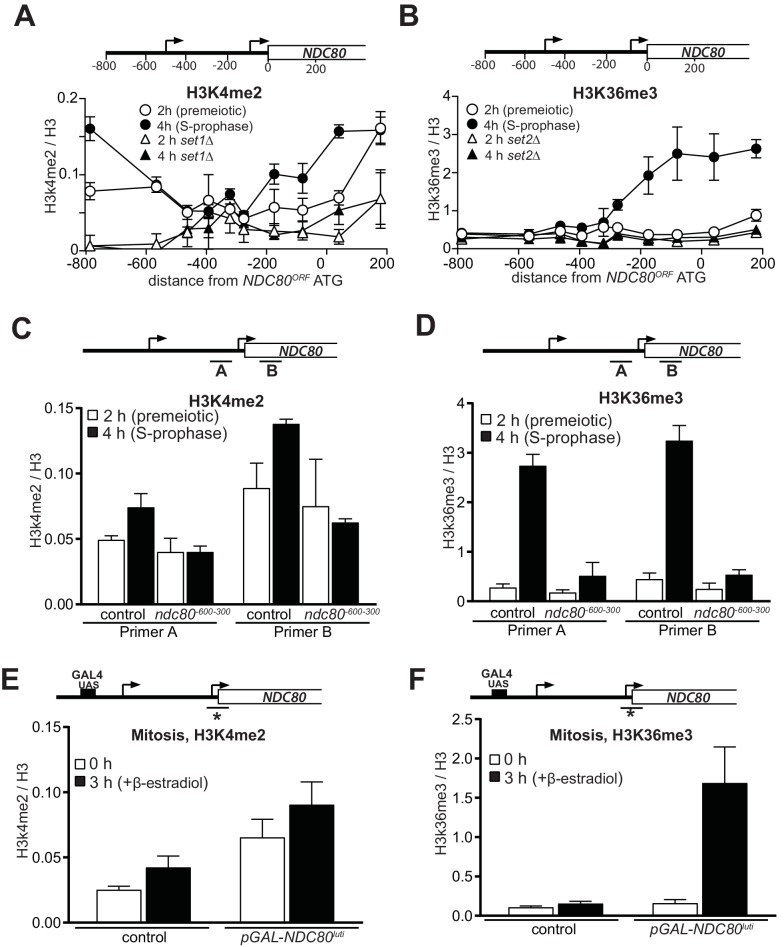
Transcription of *NDC80^luti^* promotes H3K4me2 and H3K36me3 in the promoter and 5’ region of *NDC80^ORF^*. (**A**) *NDC80^luti^* transcription promotes histone H3 lysine 4 dimethylation (H3K4me2) in the *NDC80^ORF^* promoter. Wild-type (FW1902) and *set1Δ* (FW3033) cells harboring the *pCUP-IME1*/*pCUP-IME4* were induced to undergo meiosis synchronously (see Materials and methods for details). Samples for chromatin immunoprecipitation were taken at two hours (2 hr (premeiotic), no *NDC80^luti^* transcription) and four hours in sporulation medium (SPO) (4 hr (S + prophase), *NDC80^luti^* transcription). Cells were fixed with formaldehyde, chromatin extracts were prepared and H3K4me2 or H3 enriched fragments were immunoprecipitated using magnetic Prot A beads coupled with anti-H3K4me2 or anti-H3 antibodies, respectively. The recovered DNA fragments were quantified by qPCR using ten different primer pairs scanning the *NDC80* locus. The midpoint position of each primer pair is indicated in the x-axis. The mean enrichment from three independent experiments plus the standard error of the mean for each primer pair is displayed. The H3K4me2 signal was normalized over histone H3. (**B**) Similar to A, except that histone H3 lysine 36 trimethylation (H3K36me3) abundance was determined by ChIP. Wild-type (FW1902) and *set2Δ* (FW1472) cells harboring the *pCUP-IME1*/*pCUP-IME4* alleles were used for the analysis. (**C**) Similar to A except that the ChIP for H3K4me2 was performed in control cells (FW1902) and cells harboring a deletion upstream in the *NDC80* promoter region (*ndc80^600-300^*, FW1868). For the analyses we used primer pairs directed against the *NDC80^ORF^* promoter (**A**), and the 5’ region of the *NDC80* gene (**B**). The mean fold enrichment from three independent experiments plus the standard error of the mean for each primer pair are displayed. The signals were normalized to the levels of H3. (**D**) Similar to C except that H3K36me3 levels were determined by ChIP. (**E**) Transcription of *NDC80^luti^* during mitotic growth leads to increased H3K4me2 levels in the *NDC80^ORF^* promoter. Control cells (UB91) and cells harboring *NDC80^luti^* driven by the *GAL1-10* promoter (*pGAL-NDC80^luti^*) (UB3338) were grown to exponential phase in rich medium with raffinose and galactose (YP-RG). Subsequently, cells were treated with β-estradiol, which induces translocation of the Gal4-ER chimeric transcription factor to the nucleus to activate the *GAL1-10* promoter. Samples were taken at 0 and 3 hr after induction of *NDC80^luti^* for ChIP. H3K4me2 and histone H3 levels were determined as described in C, except using a primer pair directed against the *NDC80^ORF^* core promoter. The mean enrichment from three independent experiments plus the standard error of the mean are displayed. (**F**) Similar to E except that H3K36me3 levels were determined by ChIP.

Co-transcriptional recruitment of chromatin modifying enzymes regulates the chromatin state of genes in the wake of elongating RNA polymerase II. For example, repressive chromatin marks, such as histone three lysine 4 dimethylation (H3K4me2) and lysine 36 trimethylation (H3K36me3) are deposited co-transcriptionally within gene bodies by the Set1 and Set2 methyltransferases (Hampsey and Reinberg, 2003; Kim and Buratowski, 2009). The histone deacetylase complexes Set3C and Rpd3S recognize H3K4me2 and H3K36me3, respectively, and repress cryptic transcription from chromatin carrying these modifications (Carrozza et al., 2005; Keogh et al., 2005; Kim and Buratowski, 2009; Govind et al., 2010). Set1/Set3C and Set2/Rpd3S have also been implicated in transcription-coupled repression of gene promoters (Houseley et al., 2008; Kim et al., 2012; van Werven et al., 2012; Ard and Allshire, 2016a). To investigate whether *NDC80^luti^* mediated repression of *NDC80^ORF^* also requires Set1/Set3C and Set2/Rpd3S, we measured the distribution of H3K4me2 and H3K36me3 marks at the *NDC80* locus (Figure 2A and B). We observed almost no enrichment in the *NDC80^ORF^* promoter of either marks in premeiotic cells (labelled ‘2 hr’ in Figure 2A and B), but H3K36me3 and to a lesser extent, H3K4me2 increased at the *NDC80^ORF^* promoter in meiotic prophase cells (labeled ‘4 hr’ in Figure 2A and B). As expected, the enrichment of H3K4me2 and H3K36me3 depended on Set1 and Set2, respectively (Figure 2A and B, *set1∆* and *set2∆*). In *ndc80^-600-300^* cells that do not express *NDC80^luti^*, the deposition of the H3K36me3 and H3K4me2 marks in the *NDC80^ORF^* promoter was reduced (Figure 2C and D). Thus *NDC80^luti^* transcription promotes the deposition of repressive H3K4me2 and H3K36me3 marks within the *NDC80^ORF^* promoter.

Next, we examined whether deposition of the H3K36me3 and H3K4me2 marks was dependent on the identity of the promoter that makes *NDC80^luti^* mRNA. We replaced the *NDC80^luti^* promoter with an inducible *GAL1-10* promoter, and expressed *NDC80^luti^* in rich nutrient conditions in cells harboring the Gal4-ER chimeric transcription factor, which responds to β-estradiol. We observed a moderate increase of H3K4me2 in cells that harbored *NDC80^luti^* compared to control cells, which was independent of *NDC80^luti^* transcription (Figure 2E). One explanation is that the *GAL1-10* promoter is leaky and can increase H3K4me2 levels without induction with β-estradiol. In contrast to H3K4me2, H3K36me3 levels were strongly enriched in the *NDC80^ORF^* promoter when *NDC80^luti^* was induced (Figure 2F). Control cells harboring the wild-type *NDC80^luti^* promoter did not show increased H3K36me3 levels. We conclude that the deposition of H3K36me3, but not H3K4me2, is independent of the identity of the promoter that directs *NDC80^luti^* transcription. Taken together, deposition of repressive chromatin marks in the *NDC80^ORF^* promoter requires *NDC80^luti^* transcription.

### Set2 and Set3 contribute to *NDC80^luti^* mediated repression of *NDC80^ORF^*

**Figure 3.**
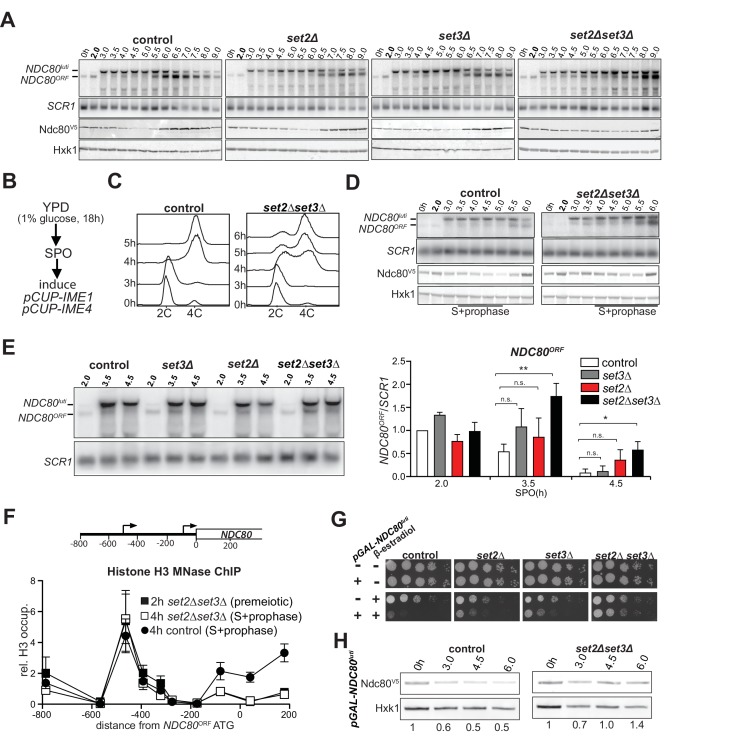
Set2 and Set3 mediate *NDC80^luti^* induced gene repression of *NDC80^ORF^*. (**A**) Set2 and Set3 are required for repression of *NDC80^ORF^* expression during early meiosis. Control (FW1902), *set2Δ* (FW2929)*, set3Δ* (FW2928) and *set2Δset3Δ* (FW1922) cells harboring *pCUP-IME1*/*pCUP-IME4* and *NDC80-V5* were grown in rich medium, transferred to pre-sporulation medium, and then shifted to SPO medium. After 2 hr, *IME1* and *IME4* expression were induced, and samples for northern and western blot analyses were taken at the indicated time points. Northern blot membranes were prepared and hybridized with a probe that detects both *NDC80^luti^* and *NDC80^ORF^* transcripts. As a loading control, membranes were also hybridized with *SCR1*. Ndc80 protein was detected with anti-V5 antibodies and Hxk1 levels were determined with anti-hexokinase antibodies. (**B**) Scheme of the synchronous meiosis protocol in which cells were shifted directly from rich medium to SPO medium. Cells were grown in rich medium (YPD) to OD_600_ of 1–2, shifted to reduced glucose medium (YPD, 1% glucose) grown overnight to saturation, and then transferred to SPO. After 2 hr, *IME1* and *IME4* were induced. (**C**) Flow cytometry analysis of DNA content in control (FW1902) and *set2Δset3Δ* (FW1922) strains. Synchronous meiosis was induced as described in B. Samples were taken at the indicated time points after transfer to SPO and were stained with propidium iodide. (**D**) Similar to A except that meiosis was induced as described in B. (**E**) Strains described in A were grown to undergo a synchronous meiosis as described in B, and selective time points were taken for northern blot analysis of *NDC80^luti^* and *NDC80^ORF^* transcripts on the same membrane. As a loading control, the northern membranes were hybridized with *SCR1*. The *NDC80^ORF^* levels were quantified (right panel) and data from three independent experiments plus the standard error of the mean (SEM) is displayed. One-tailed, unpaired t-tests were conducted to test if the differences in *NDC80^ORF^* levels were statistically significant. A single asterisk * denotes p-value<0.05. A double asterisk ** denotes p-value<0.01. ‘n.s.’ means ‘not significant’. To control for technical variation between different northern blots, the *NDC80^ORF^* signal from the two hour time point from the control strain of each blot was set to one. (**F**) *NDC80^luti^* transcription requires Set2 and Set3 to establish a repressive chromatin state at the promoter of *NDC80^ORF^.* Chromatin structure at the *NDC80* locus was determined by ChIP of histone H3 on micrococcal nuclease (MNase) treated extracts in control (FW1902) and *set2Δ set3Δ* (FW1922) cells as described in A. Samples were taken prior to *IME1*/*IME4* induction at 2 hr in SPO (2 hr, premeiotic) and after induction at 4 hr in SPO (4 hr, S + prophase), fixed with formaldehyde, and mononucleosome fragments were isolated. The recovered DNA fragments were quantified by qPCR using ten different primer pairs directed against the *NDC80* locus relative to a no MNase input. The signals from each primer pair were then normalized over a primer pair directed against the *PHO5* core promoter. The midpoint position of each primer pair is indicated in the x-axis. The mean signal from three independent experiments plus the standard error of the mean for each primer pair is displayed. (**G**) Ectopic expression of *NDC80^luti^* is lethal in mitosis, but is rescued in a *set2Δ set3Δ* mutant. Spot assays of control cells, which harbor a wild-type *NDC80* locus, with *SET2 SET3* (UB1252), *set2Δ* (UB3545), *set3Δ* (UB3547), and *set2Δ set3Δ* (UB3549); as well as cells expressing *NDC80^luti^* from the heterologous *GAL* promoter (*pGAL-NDC80^luti^*) with *SET2 SET3* (UB1218), *set2Δ* (UB1236), *set3Δ* (UB1237), and *set2Δ set3Δ* (UB1235). These cells also expressed the Gal4 fused to estrogen receptor (Gal4-ER), which translocates to the nucleus in the presence of β-estradiol to activate the *GAL1-10* promoter. Cells were grown overnight on YP-glycerol plates, diluted in sterile water, and spotted on YP +raffinose + galactose (YP-RG) plates in the absence or presence of β-estradiol. (**H**) Ectopic expression of *NDC80^luti^* fails to downregulate Ndc80 in the *set2Δ set3Δ* mutant. Cells expressing *NDC80^luti^* from the *GAL* promoter with *SET2 SET3* (UB1217) or *set2Δ set3Δ* (UB8114) were grown to exponential phase in YP-RG, and they were induced to express *NDC80^luti^* with β-estradiol. Samples were taken at the indicated time points. Ndc80 protein levels were determined by western blot using anti-V5 antibodies. Hxk1 levels were detected with anti-hexokinase antibodies. Ndc80 and Hxk1 were quantified and the relative expression (Ndc80/Hxk1) with respect to the 0 hr time point is displayed.

Because H3K36me3 and H3K4me2 marks localize to the *NDC80^ORF^* promoter when *NDC80^luti^* is transcribed, we examined whether Set1/Set3C and Set2/Rpd3S contribute to *NDC80^ORF^* repression. Since Set1 also plays an important role in meiotic recombination, we deleted *SET3* to test how the Set1/Set3C pathway regulates the *NDC80* locus (Borde et al., 2009; Acquaviva et al., 2013; Sommermeyer et al., 2013). In the *set2∆ set3∆* double mutant, but not the single mutants, both *NDC80^luti^* and *NDC80^ORF^* transcripts were detected throughout multiple time points in early meiosis, and the steady-state level of Ndc80 protein remained high (Figure 3A and Figure 3—figure supplement 1A, compare the time points from two to five hours between control and mutant cells). The *set2∆ set3∆* double mutant cells entered and underwent meiosis with delayed kinetics (Figure 3—figure supplement 1B). Thus, it is possible that a population of cells never entered meiosis and continued to express the mitotic *NDC80^ORF^* mRNA isoform. We improved the kinetics of meiosis by adopting a different meiotic synchronization protocol (Figure 3B). Instead of growing cells in pre-sporulation medium, we shifted them directly to sporulation medium after they reached saturation in nutrient rich conditions. We then induced *IME1* and *IME4*. This synchronization procedure reduced the delay in meiotic divisions (compare Figure 3—figure supplement 1B and C). In addition, meiotic S phase was completed in more than 75 percent of cells after 6 hr, indicating that the majority of cells had entered meiosis (Figure 3C). Importantly, *NDC80^luti^* mediated repression was still compromised in *set2∆ set3∆* double mutant cells despite improved synchrony of meiosis (Figure 3D and Figure 3—figure supplement 1D, compare the time points from three-to five hours for the control with three-to six hours for the mutant cells). Further analyses of selective time-points (3.5 and 4.5 hr) confirmed that there were significant differences in *NDC80^ORF^* levels between the control and the *set2∆ set3∆* double mutant, but not the single mutants (Figure 3E).

**Figure 3—figure supplement 1.**
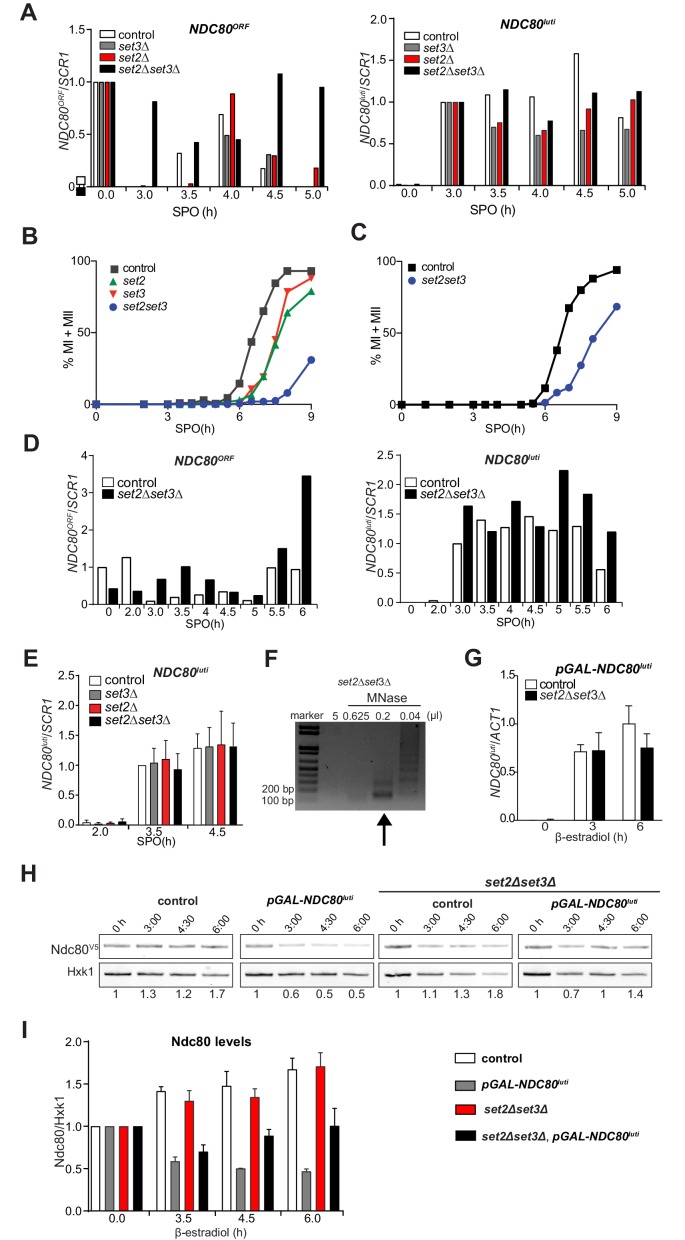
The Set2 and the Set3 mediate *NDC80^luti^* induced gene repression of *NDC80^ORF^*. (**A**) Quantification of *NDC80^ORF^* and *NDC80^luti^* levels in the experiment described in Figure 3A. Signals are normalized to *SCR1.* To control for variation in overall signal between different northern blots, the *NDC80^ORF^* signal at the 0 hr time point was set to one, and the *NDC80^luti^* signal at the 3 hr time point was set to one. (**B**) *set2Δ set3Δ* mutants undergo meiosis with delayed kinetics. Kinetics of meiotic divisions (MI + MII) in control (FW1902), *set2Δ* (FW2929)*, set3Δ (*FW2928) and *set2Δset3Δ* (FW1922) cells harboring *pCUP-IME1*/*pCUP-IME4* and *NDC80-V5.* Cells were grown in rich medium, transferred to pre-sporulation medium, and then shifted to sporulation medium. After 2 hr, *IME1* and *IME4* expression were induced, and samples were taken at the indicated time points, fixed, and stained with DAPI. The percentage of cells with one, two or more DAPI masses was determined for at least 200 cells per time point. (**C**) Similar to B, except that cells were directly shifted from rich medium to SPO. (**D**) Quantification of *NDC80^ORF^* and *NDC80^luti^* levels in the experiment described in Figure 3D. Signals are normalized to *SCR1.* The relative expression (*NDC80^ORF^/SCR1*) with respect to the 0 hr time point is displayed (left panel). The relative expression (*NDC80^luti^*/*SCR1*) with respect to the 3 hr time point is displayed (right panel). (**E**) Same experiment as Figure 3E, except that the *NDC80^luti^* levels on northern blots were quantified, first normalized to *SCR1* and then normalized to the intensity of *NDC80^luti^* at 3.5 hr in control cells. The mean from three independent experiments plus the standard error of the mean are displayed. (**F**) Example of extract with mononucleosomes from *set2Δset3Δ* cells (FW1922, 2 hr [premeiotic]) prepared as described in Materials and methods. In short, cells were fixed with formaldehyde, treated with zymolase, and subsequently treated with different concentrations of micrococcal nuclease (MNase). Part of the sample from each extract was reverse crosslinked, purified, and separated by gel electrophoresis. The arrow indicates the extract that was used for subsequent ChIP analysis. (**G**) Levels of *NDC80^luti^* are not affected in the *set2Δ set3Δ* double mutant. Control (UB1217) and *set2Δ set3Δ* (UB8114) cells harboring *NDC80^luti^* driven by the *GAL1-10* promoter were grown in YP-RG medium overnight, diluted and treated with β-estradiol during exponential growth. Samples were taken at the indicated time points. RNA was extracted, reverse transcribed, and the *NDC80^luti^* mRNA levels were determined by qPCR. Signals were normalized to *ACT1*. The mean from three independent experiments plus the standard error of the mean are displayed. (**H**) Ectopic expression of *NDC80^luti^* fails to downregulate Ndc80 in the *set2Δ set3Δ* mutant. Western blot of Ndc80 for *SET2 SET3* cells harboring an *NDC80-V5* allele (UB1240) or harboring a *pGAL-NDC80^luti^-V5* allele (UB1217), as well as *set2Δset3Δ* cells with *NDC80-V5* (UB8110) or *pGAL-NDC80^luti^-V5* (UB8114). Cells were grown as described in Figure 3H, treated with β-estradiol, and samples were taken at the indicated time points. Ndc80 protein levels were determined with anti-V5 antibodies. Hxk1 levels were detected with anti-hexokinase antibodies. Ndc80 and Hxk1 were quantified, and the relative expression (Ndc80/Hxk1) with respect to the 0 hr time point is indicated. The western blot is from the same experiment described in Figure 3H, except that the data for the strains harboring the *NDC80-V5* allele are included. (**I**) Quantification of Figure 3—figure supplement 1H. Ndc80 signals were first normalized to Hxk1 and were then normalized with respect to the 0 hr time point. The mean from three independent experiments plus the standard error of the mean are displayed.

Previous work showed that the *set2∆* mutant exhibits increased nucleosome dynamics leading to de-repression of cryptic promoters (Venkatesh et al., 2012). In addition, the *set3∆* mutant displays reduced histone H3 density in the 5’ region of transcribed genes (Kim and Buratowski, 2009). Set2 and Set3 are also required for transcription coupled chromatin changes in the *IME1* promoter by the long noncoding RNA *IRT1* (van Werven et al., 2012). These findings prompted us to examine whether Set2 and Set3 are necessary for *NDC80^luti^* mediated nucleosome assembly in the *NDC80^ORF^* promoter. We found that even though *NDC80^luti^* was efficiently transcribed in *set2∆ set3∆* cells during early meiosis (Figure 3E, Figure 3—figure supplement 1D, right panel, and Figure 1E), repressive chromatin was not established at the *NDC80^ORF^* promoter (Figure 3F and Figure 3—figure supplement 1F).

We also tested whether Set2 and Set3 are required for repression of *NDC80^ORF^* when *NDC80^luti^* is expressed ectopically during vegetative growth. Although *NDC80^luti^* transcription was lethal in wild-type control cells, there was a partial growth rescue in either *set2∆* or *set3∆* cells. Importantly, growth was restored almost completely in *set2∆ set3∆* cells (Figure 3G), due to de-repression of Ndc80 protein expression despite elevated transcription of the *NDC80^luti^* mRNA (Figure 3H, Figure 3—figure supplement 1G–I). Altogether, these results show that both Set2 and Set3 are necessary for efficient repression of the *NDC80^ORF^* promoter by *NDC80^luti^* transcription.

### Gene repression by *NDC80^luti^* is reversible

**Figure 4.**
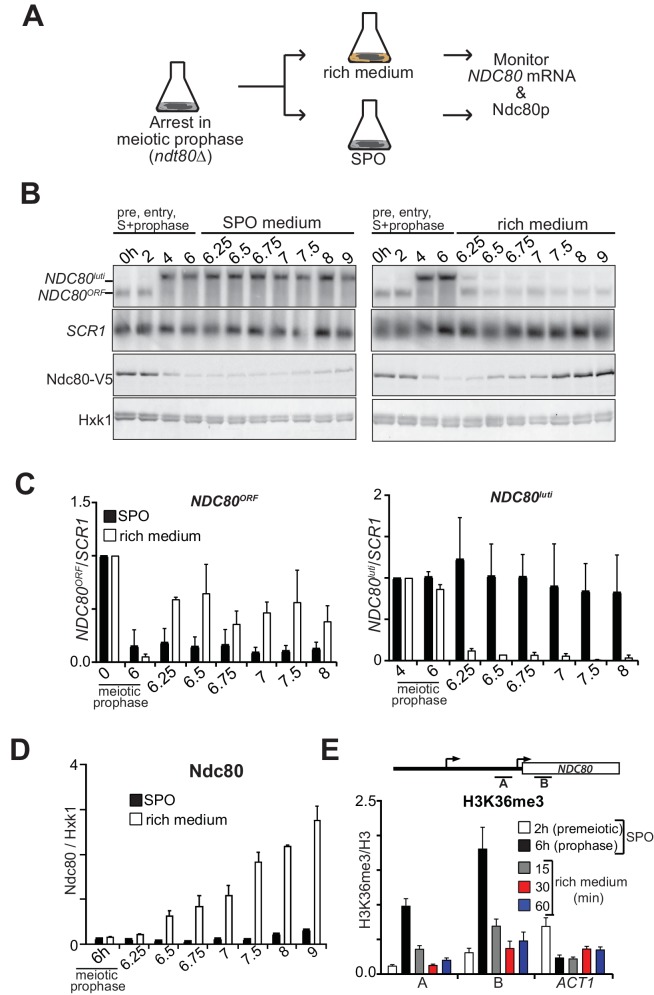
*NDC80^luti^* mediated repression is reversible. (**A**) Schematic overview of the experimental set-up. (**B**) Cells repress *NDC80^luti^* and induce *NDC80^ORF^* when returned to a nutrient rich environment (YPD). Cells harboring *ndt80Δ* and *pCUP-IME1/pCUP-IME4* (FW3856) were grown in rich medium, shifted and grown in pre-sporulation medium, and subsequently transferred to SPO. Samples were taken prior to induction of *IME1* and *IME4* at 0 hr and 2 hr (premeiotic [pre]), and after induction at 4 hr (S-phase) and 6 hr (prophase) in SPO. After 6 hr, cells were either transferred to rich medium or kept in SPO medium. Samples for RNA and protein were taken at the indicated time points. To detect *NDC80^luti^* and *NDC80^ORF^* expression, RNA was extracted, separated by gel electrophoresis, blotted, and hybridized with a probe that spans the *NDC80* promoter and coding region. As a loading control for northern blots, we also probed membranes for *SCR1*. Ndc80 protein levels were determined by western blot using anti-V5 antibodies. As a loading control we also detected Hxk1 levels with anti-Hxk1 antibodies. (**C**) Quantification of *NDC80^ORF^* and *NDC80^luti^* levels as described in B. The signals were normalized over *SCR1*. The error bars represent the standard error of the mean from two independent experiments. To control for technical variation between experiments the 0 hr and 4 hr time points were set to one for *NDC80^ORF^* and *NDC80^luti^*, respectively. (**D**) Quantification of Ndc80 protein levels during return to rich medium as described in B. The Ndc80 protein levels were normalized to Hxk1 protein abundance. The relative levels with respect to the 6 hr time point are displayed. The mean from two independent experiments plus the standard error of the mean is displayed. (**E**) H3K36me3 is rapidly lost from the *NDC80^ORF^* promoter and 5’ region after return to growth. Growth conditions were similar to B, except that histone H3 lysine 36 trimethylation (H3K36me3) levels were quantified at the *NDC80^ORF^* promoter during return to growth in a nutrient rich environment. Samples for chromatin immunoprecipitation were taken at the indicated time points. Cells were fixed with formaldehyde, chromatin extracts were prepared and H3K36me3 and histone H3 enriched fragments were immunoprecipitated with anti-H3K36me3 or anti-H3 antibodies, respectively. The recovered DNA fragments were quantified by qPCR using a primer directed against the *NDC80^ORF^* promoter (primer A) and a primer directed against the 5’ region of the *NDC80* gene (primer B). The mean enrichment from three independent experiments plus the standard error of the mean for each primer pair is displayed. We also analyzed the signal at the 3’ end of the *ACT1* open reading frame. The H3K36me3 signals were normalized to the histone H3 signal.

Ndc80 is an essential kinetochore protein required for chromosome segregation in both mitosis and meiosis (Ciferri et al., 2007). Thus, Ndc80 levels must be restored when cells either transit from prophase to meiotic divisions or when cells re-enter the mitotic cycle before meiotic commitment. This transition necessitates that the repression by *NDC80^luti^* transcription be rapidly reversible so that cells can resume cell divisions in a timely manner. Indeed, *NDC80^ORF^* levels swiftly increase prior to meiotic divisions to facilitate chromosome segregation (Chen et al., 2017). To examine how gene repression by *NDC80^luti^* transcription can adapt to changes in cell fate, we measured *NDC80^luti^* and *NDC80^ORF^* expression kinetics in meiotic prophase cells that re-entered the mitotic cell cycle (Figure 4A). Cells carrying the *ndt80∆* mutation were arrested in meiotic prophase, and returned to growth by providing them with rich medium. Strikingly, almost no *NDC80^luti^* mRNA could be detected 15 min after return to growth (Figure 4B–C and Figure 4—figure supplement 1A). Concomitantly, *NDC80^ORF^* was almost fully re-expressed at the same time and Ndc80 protein levels increased after 30 min (Figure 4B–D). Therefore, *NDC80^luti^* mediated repression of *NDC80^ORF^* is rapidly reversible, allowing for adaptation to physiological needs and re-entry into the mitotic cell cycle.

**Figure 4—figure supplement 1.**
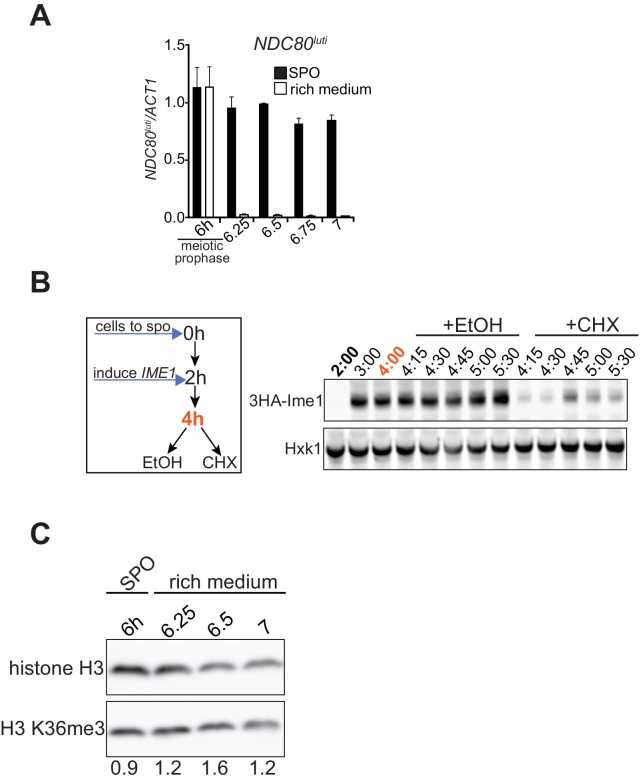
*NDC80^luti^* mediated repression is reversible. (**A**) Quantification of *NDC80^luti^* during return to rich medium as described in Figure 4B. RNA was extracted, reverse transcribed, and *NDC80^luti^* mRNA levels were determined by quantitative PCR. Signals were normalized to *ACT1*. The mean from two independent experiments plus the standard error of the mean is displayed. (**B**) Ime1 protein is unstable. Cells harboring Hemagglutinin-tagged *IME1* under the control of the *CUP1* promoter were grown in rich medium (UB1026), shifted to pre-sporulation medium, and subsequently transferred to SPO. After 2 hr, Ime1 was induced. After 4 hr in SPO, cells were split, and either treated with ethanol (EtOH) or cycloheximide (CHX, 0.2 mg/ml). Samples were taken at the indicated time points. Ime1 protein levels were determined by western blot using anti-HA antibodies. As a loading control, we also detected Hxk1 levels. (**C**) Bulk histone H3K36me3 levels remain constant during return to a nutrient rich environment. Cells harboring *ndt80Δ* (FW3856) were grown in rich medium, shifted and grown in pre-sporulation medium, and subsequently transferred to SPO. After 6 hr, cells were either transferred to rich medium or continued in SPO medium. Samples were taken at the indicated time point. H3K36me3 and histone H3 levels were determined by western blot using anti-H3K36me3 and anti-histone H3 antibodies, respectively. The relative abundance of H3K36me3 normalized to histone H3 is indicated.

One explanation for the swift shut down of *NDC80^luti^* transcription is that its transcriptional activator is degraded or depleted. The accompanying paper showed that Ime1 is the major transcriptional activator of *NDC80^luti^* transcription (Chen et al., 2017). Under nutrient rich conditions, *IME1* transcription shuts down and Ime1 protein translocates to the cytoplasm (Colomina et al., 2003; van Werven and Amon, 2011). In addition, we found that Ime1 has a half-life of only a few minutes, facilitating rapid clearance of Ime1 protein during return-to-growth (Figure 4—figure supplement 1B). We propose that during return-to-growth, Ime1 is rapidly inactivated, resulting in a quick decrease in *NDC80^luti^* transcription.

We hypothesized that the dynamic changes of *NDC80^luti^* mediated repression during return to growth would be reflected in the chromatin state of the *NDC80^ORF^* promoter. We found that H3K36me3 at the 5’ end of *NDC80^ORF^* was strongly reduced within 15 min and almost completely lost within 30 min after cells returned to a nutrient rich environment (Figure 4E). The loss was specific to the *NDC80* locus because the levels at the 3’ end of the *ACT1* gene increased slightly while bulk H3K36me3 levels did not change (Figure 4E and Figure 4—figure supplement 1C). Thus *NDC80^luti^*-mediated gene repression is reversible, allowing for rapid and dynamic changes in gene expression and chromatin state.

### Gene repression by *NDC80^luti^* transcription is tunable

**Figure 5.**
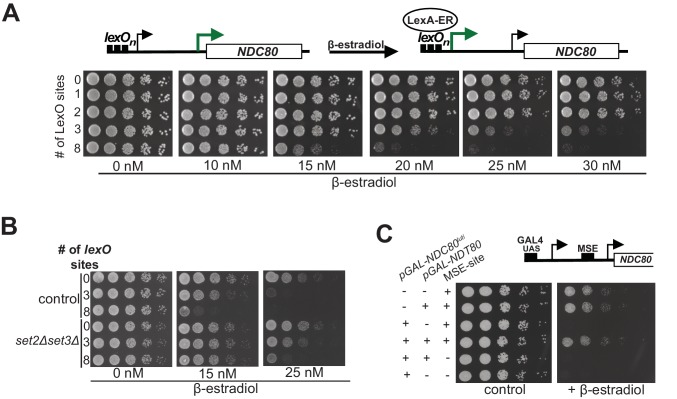
Gene repression by *NDC80^luti^* is tunable. (**A**) Adjustable expression of *NDC80^luti^* using the LexA-*lexO* system. Spot assay of cells harboring 0, 1, 2, 3, or 8 *lexO* binding sites in the *NDC80^luti^* promoter (UB8374, UB8358, UB8362, UB8366, UB8370) in the presence of different concentrations of β-estradiol. These cells also expressed LexA fused to an activation domain (AD) and the human estrogen receptor (ER) (LexA-ER-AD). Cells were grown overnight, diluted in sterile water, and spotted on YPD plates in the absence or presence of different concentrations of β-estradiol. (**B**) Similar to A, except that *set2Δset3Δ* mutant cells (UB8691, UB8686 and UB8693) were included for the analysis. (**C**) Increased *NDC80^ORF^* promoter activity bypasses *NDC80^luti^* mediated repression. Cells were grown overnight, diluted in sterile water, and spotted on YP +raffinose + galactose plates in the absence or presence of β-estradiol (1 μM). For the analyses, we used three sets of strains: (1) Cells with a wild-type *NDC80* and with a functional MSE site (+MSE), but with either a wild-type *NDT80* (UB3351) or a *pGAL1-10* driven *NDT80* (*pGAL-NDT80*, UB3370); (2) cells with *pGAL-NDC80^luti^* and with a functional MSE site, along with either a wild-type *NDT80* (UB5154) or *pGAL-NDT80* (UB9181); (3) cells with *pGAL-NDC80^luti^* and a non-functional MSE site, along with either *pGAL-NDT80* (UB9921) or wild-type *NDT80* (UB9923). These cells also expressed Gal4-ER to activate *pGAL* driven expression.

Work from *Escherichia coli* showed that gene regulation by transcriptional interference is not binary with an on or off state, but can be utilized to fine-tune gene expression levels (Bordoy et al., 2016; Hao et al., 2016). The work prompted us to investigate whether transcriptional interference by *NDC80^luti^* could also be tunable, thus enabling incremental changes in *NDC80^ORF^* expression levels. To scale the level of *NDC80^luti^* expression, we used a tightly controlled, inducible system. The system utilizes a heterologous, chimeric transcriptional activator (LexA-ER-AD) whose activity is induced in a concentration-dependent manner by β-estradiol (Ottoz et al., 2014). Varying the number of LexA-binding sites (*lexO*) in the *NDC80^luti^* promoter and titrating the concentration of β-estradiol, enabled scalable transcriptional induction of *NDC80^luti^* (Figure 5—figure supplement 1A). The growth defect caused by *NDC80^luti^* expression in mitosis was more severe with elevated concentrations of β-estradiol and higher number of *lexO* sites in the *NDC80^luti^* promoter (Figure 5A). The higher the *luti* transcription, the greater the inhibition of *NDC80^ORF^* expression. Thus, modulating *NDC80^luti^* transcription levels allows scalable transcriptional repression of *NDC80^ORF^* in a population of cells.

**Figure 5—figure supplement 1.**
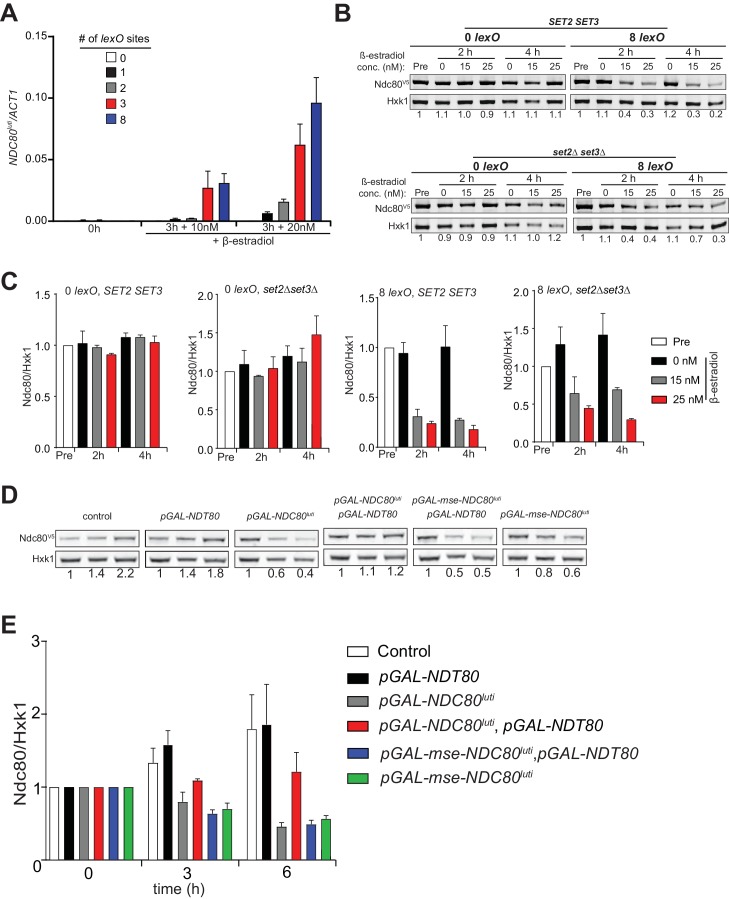
Gene repression by *NDC80^luti^* is tunable. (**A**) *NDC80^luti^* levels in the presence of variable number of *lexO* sites in the *NDC80^luti^* promoter. Cells harboring 0, 1, 2, 3, or 8 *lexO* and LexA-ER-AD (UB8374, UB8358, UB8362, UB8366, and UB8370) were grown in YPD overnight. Subsequently, cells were diluted and exponentially growing cells were treated with 10 or 20 nM β-estradiol for 3 hr. RNA was extracted, reverse transcribed, and *NDC80^luti^* mRNA levels were determined by quantitative PCR. Signals were normalized to *ACT1*. The mean from three independent experiments plus the standard error of the mean are displayed. (**B**) Ndc80 protein level in *SET2 SET3* cells harboring none (UB12945) or 8 *lexO* sites (UB12949), or *set2∆ set3∆* cells with none (UB12947) or 8 *lexO* sites (UB12951). All four strains carry LexA-ER-AD. Ndc80 protein was detected by anti-V5 immunoblot. Hxk1 levels were used as a loading control. ‘Pre’ denotes pre-induction. Exponentially growing cells were treated with ethanol (0 nM), 15 nM, or 20 nM β-estradiol. Samples were taken at 2 hr or 4 hr after β-estradiol induction. Ndc80 level was normalized to Hxk1 level, and the number under each lane shows the Ndc80/Hxk1 ratio normalized to that in the pre-induction condition. (**C**) Same as Figure 5—figure supplement 1B, except that the mean of two independent experiments plus standard error of the mean are displayed. (**D**) Increased promoter *NDC80^ORF^* activity bypasses *NDC80^luti^* mediated repression. Exponentially growing cells were treated with ethanol or 1 μM β-estradiol. Samples were taken at 3 hr or 6 hr after β-estradiol induction. The amount of samples loaded corresponded to the same OD across all the cultures. Ndc80 level was normalized to Hxk1 level, and the number under each lane shows the Ndc80/Hxk1 ratio normalized to that in the pre-induction condition. For the analyses, we used the strains described in Figure 5C. (**E**) Quantification of Ndc80 protein levels from Figure 5—figure supplement 1D. The Ndc80 protein levels were normalized to Hxk1 protein abundance. The relative levels with respect to the 0 hr time point are displayed. The mean from three independent experiments plus the standard error of the mean are displayed.

During transcription nucleosomes are disassembled and reassembled by histone chaperones that associate with RNA polymerase (Venkatesh and Workman, 2015). Therefore, higher levels of *NDC80^luti^* transcription could lead to an increased rate of nucleosome deposition in the *NDC80^ORF^* promoter and thus scalable *NDC80^ORF^* repression. If so, then sufficiently high levels of *NDC80^luti^* transcription should be sufficient for repressing *NDC80^ORF^* without requiring Set1/Set3C and Set2/Rpd3S to maintain repressive chromatin. Cells with both pathways compromised (*set2∆set3∆*) and harboring three or eight *lexO* sites did not show a growth defect when exposed to intermediate levels of β-estradiol (15 nM), whereas control cells did (Figure 5B). This result was expected because in the *set2∆set3∆* mutant background *NDC80^luti^* mediated repression is impaired (also see Figure 3). Surprisingly at higher concentrations of β-estradiol (25 nM), *set2∆set3∆* mutant cells harboring three *lexO* sites exhibited a moderate growth defect while cells with eight *lexO* sites exhibited a severe growth defect. We also measured the Ndc80 protein levels in control and *set2∆set3∆* mutant cells harboring 0 or 8 copies of *lexO* sites. The growth defects observed in Figure 5B were reflected in the Ndc80 protein levels (Figure 5—figure supplement 1B and C). These data suggest that high levels of *NDC80^luti^* transcription could bypass the requirement for Set2 and Set3 in *NDC80^ORF^* repression.

Since increased expression of *NDC80^luti^* leads to stronger repression of *NDC80^ORF^*, we tested whether the strength of the *NDC80^ORF^* promoter influenced the effectiveness of *NDC80^luti^* mediated repression. To examine this, we increased the levels of *NDC80^ORF^* by ectopically expressing the meiotic transcription factor Ndt80 in mitotic cells. Ndt80 induces the expression of *NDC80^ORF^* via the middle sporulation element (MSE) in the *NDC80^ORF^* promoter (Chen et al., 2017). In the presence of Ndt80 expression, the growth defect caused by *NDC80^luti^* transcription was suppressed (Figure 5C). This suppression is dependent on the presence of the MSE site in the *NDC80^ORF^* promoter (Figure 5C, compare MSE positive versus negative in the presence of *pGAL-NDT80* and *pGAL-NDC80^luti^*). In addition, the growth changes as observed in the spot assays correlated well with Ndc80 protein levels (Figure 5—figure supplement 1D and E). Thus, increased transcription from the *NDC80^ORF^* promoter can bypass *NDC80^luti^* mediated repression. Taken together, transcriptional interference by expression of a 5’ extended transcript can be tuned by adjusting the relative strengths of the distal and proximal promoters. Hence, this mechanism can be adapted as a regulatory module to generate a range of gene expression outputs.

## Discussion

Eukaryotic cells have evolved various mechanisms to achieve dynamic control of gene expression during cell fate progression. Here we demonstrate how Ndc80 levels are temporally regulated during the budding yeast meiotic program. Together with an accompanying paper, we show that *NDC80* transcription is repressed by an unusual mechanism during the prophase stage of meiosis (Chen et al., 2017). Transcription of a 5’ extended mRNA isoform interferes with transcription of the downstream canonical *NDC80* mRNA isoform. The mechanism of gene repression described here is tunable, rapidly reversible, and an effective way to generate changes in gene expression.

### The transcriptional mechanism of *NDC80* repression

Repression of Ndc80 protein production during meiotic prophase is critical for setting up faithful chromosome segregation during meiosis (Miller et al., 2012). Two *NDC80* mRNA isoforms were identified: the Ndc80 coding isoform (*NDC80^ORF^*), and the 5’ extended long undecoded transcript isoform (*NDC80^luti^*), which cannot be translated into Ndc80 protein (Chen et al., 2017). We showed that *NDC80^luti^* transcription interferes with the downstream *NDC80^ORF^* promoter. First, the expression of *NDC80^luti^* and *NDC80^ORF^* are anti-correlated in cells undergoing meiosis synchronously. Second, mutants defective in *NDC80^luti^* transcription cannot repress *NDC80^ORF^* (Chen et al., 2017). In particular transcription of truncated *NDC80^luti^* fails to repress *NDC80^ORF^*, thus excluding promoter competition as a mechanism for repression. Third, transcription of *NDC80^luti^* inhibits TFIIB binding to the *NDC80^ORF^* promoter (Figure 1). Fourth, repression by *NDC80^luti^* only works in *cis* and *NDC80^luti^* RNA does not localize to a discrete region in the nucleus, making it unlikely that the *NDC80^luti^* RNA itself is important to the repression mechanism (Chen et al., 2017). However, we cannot rule out that the nascent *NDC80^luti^* RNA contributes to repression of *NDC80^ORF^* expression.

**Figure 6.**
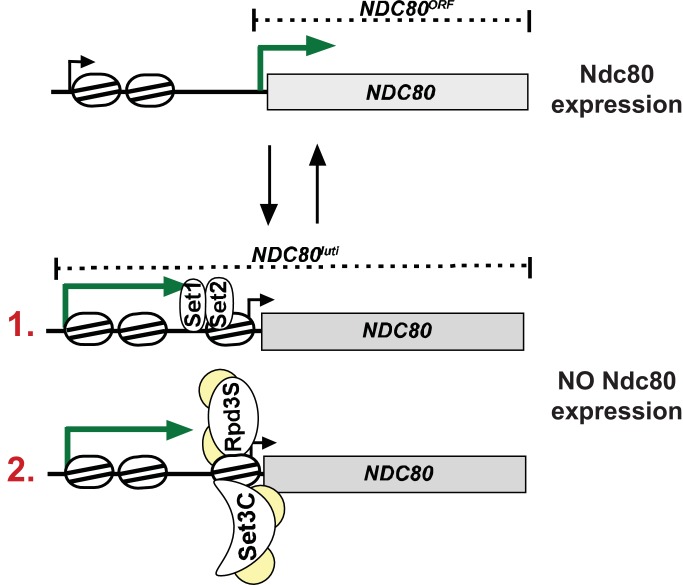
Model for *NDC80^luti^* mediated repression of *NDC80^ORF^*. During mitosis the *NDC80^ORF^* promoter has an open chromatin structure and *NDC80^ORF^* is transcribed. During meiotic S-phase and meiotic prophase, transcription of *NDC80^luti^* deposits Set1-dependent H3K4me2 and Set2-dependent H3K36me3 in the same area of the *NDC80^ORF^* promoter. Co-transcriptional deposition of these marks facilitates the recruitment of the histone deacetylase complexes Set3C and Rpd3S. This leads to increased nucleosome occupancy in the *NDC80^ORF^* promoter and *NDC80^ORF^* transcription is repressed. The set of reversing arrows indicates that *NDC80^luti^* mediated repression of *NDC80^ORF^* is reversible.

How does *NDC80^luti^* interfere with *NDC80^ORF^* transcription? Our data show that Set2 and Set3 is essential for establishing a repressive chromatin state and for inhibiting *NDC80^ORF^* transcription. We propose a two-step mechanism (Figure 6). First, transcription of *NDC80^luti^* deposits Set1 mediated H3K4me2 and Set2 mediated H3K36me3 in the *NDC80^ORF^* promoter. Second, co-transcriptional deposition of these marks facilitates the recruitment of the histone deacetylase complexes Set3C and Rpd3S (Carrozza et al., 2005; Keogh et al., 2005; Kim and Buratowski, 2009). Notably, the two marks localize to the same area of the *NDC80^ORF^* promoter, perhaps indicating that there is redundancy between the two pathways (see following section in the discussion). Previous work established a role for Set2 in suppressing histone exchange and promoting nucleosome stability through chromatin remodelers (Venkatesh et al., 2012; Smolle et al., 2012). Hypo-acetylated histones are also associated with increased nucleosome stability (Venkatesh and Workman, 2015). Hence, cells lacking both Set2 and Set3 show reduced nucleosome occupancy in the *NDC80^ORF^* promoter, and *NDC80^ORF^* transcription is unimpeded despite active *NDC80^luti^* transcription (Figure 3). Taken together, *NDC80^luti^* repression of *NDC80^ORF^* is mediated by co-transcriptional chromatin reorganization of the *NDC80^ORF^* promoter.

The Set1/Set3C and Set2/Rpd3S pathways have well characterized roles in preventing cryptic transcription and regulating gene expression via long noncoding RNA transcription (Carrozza et al., 2005; Keogh et al., 2005; Kim and Buratowski, 2009; Kim et al., 2012; van Werven et al., 2012; Ard and Allshire, 2016b ; Venkatesh et al., 2016). It has been reported that Set2 and Set3 modulate the expression of different genes based on the length of adjacent ncRNAs, which overlap with their promoters. (Kim et al., 2012; Kim et al., 2016). *Kim et al.* demonstrate that during a series of carbon source shifts, genes whose promoters overlap with longer transcripts (~2.0 kb) are repressed by Set2/Rpd3S whereas those with shorter overlapping transcripts (~0.9 kb), are repressed by Set1/Set3C (Kim et al., 2016). According to their classification, *NDC80^luti^* is a short overlapping transcript. Strikingly, *NDC80^luti^* mediated repression of *NDC80^ORF^* was compromised in the *set2∆set3∆* double mutant cells, but not in the single mutants. (Figure 3). We propose that Set1/Set3C and Set2/Rpd3S act redundantly during *NDC80^luti^* mediated repression of *NDC80^ORF^*.

The mechanism that we have described here has clear similarities with transcriptional interference mediated by intergenic or promoter transcription. Like *NDC80^luti^*, transcription of the intergenic/promoter ncRNA, *SRG1*, leads to increased nucleosome occupancy and lower binding of activators in the downstream *SER3* promoter (Martens et al., 2004; Hainer et al., 2011). Transcriptional interference is also important for mating-type control of sporulation. In cells with a single mating type, transcription of a long noncoding RNA *IRT1*, in the promoter of the master regulatory transcription factor Ime1, is critical for *IME1* repression (van Werven et al., 2012). Similar to *NDC80^luti^*, *IRT1* transcription establishes repressive chromatin throughout the *IME1* promoter via a mechanism requiring both Set2 and Set3. Interestingly, while Set1 and Set2 act on different parts of the *IME1* promoter, the H3K4me2 and H3K36me3 marks overlap in the *NDC80^ORF^* promoter suggesting that both modifications control the same promoter region. Perhaps, H3K4me2 and H3K36me3 occur on the same nucleosome as part of a repressive combinatorial histone code.

### *NDC80^luti^* mediated repression of *NDC80^ORF^* is dynamic

Our synchronous meiosis and return to growth experiments have shed light on the dynamics of *NDC80^luti^* mediated repression of *NDC80^ORF^*. During meiotic prophase Ndc80 levels decline, but as soon as cells enter meiotic divisions, Ndc80 levels increase. *Chen et al.* showed that the transcription factor Ndt80 activates the meiotic wave of *NDC80^ORF^* expression (Chen et al., 2017). Expression of Ndt80 can bypass *NDC80^luti^* induced repression suggesting that repression is not refractory to increasing levels of *NDC80^ORF^* transcription (Figure 5). This ensures that *NDC80^ORF^* can be rapidly produced after meiotic prophase, when Ndc80 becomes essential for proper meiotic divisions. Interestingly, the MSE site (approximately −200 bp upstream from the AUG) in the *NDC80^ORF^* promoter is not protected by nucleosomes even during *NDC80^luti^* transcription (Figure 1E), which may explain the ability of Ndt80 to activate *NDC80^ORF^* even while *NDC80^luti^* is expressed.

The dynamic nature of the regulation is also illustrated by our finding that *NDC80^luti^* mediated repression can be rapidly reversed (Figure 4). We propose that the reversibility of the repression may provide two main benefits. First, it allows for temporal control of *NDC80^ORF^* expression during meiosis as *NDC80^luti^* mediated repression of *NDC80^ORF^* specifically occurs during meiotic S-phase and meiotic prophase. Cells that progressed into meiotic divisions were able to bypass *NDC80^luti^* mediated repression and re-expressed *NDC80^ORF^* (Chen et al., 2017). Second, rapid adjustments to *NDC80* expression in response to changing environmental cues can be directly integrated at the *NDC80* promoter. When we exposed cells arrested in meiotic prophase to a nutrient rich environment and allowed cells to re-enter the mitotic cell cycle, *NDC80^luti^* expression was lost and *NDC80^ORF^* was rapidly induced (Figure 4). The mechanism for the reversibility of *NDC80^luti^* mediated repression is not fully understood. We speculate that transcriptional activators and chromatin remodelers stimulate nucleosome eviction in the *NDC80^ORF^* promoter during its activation. In line with this idea, levels of the repressive H3K36me3 in the *NDC80^ORF^* promoter are rapidly lost when the repression is reversed (Figure 4E). This is despite the purported stability of the H3K36me3 mark due to its role in reducing histone turnover (Smolle et al., 2012; Venkatesh et al., 2012; Sein et al., 2015). More work is needed to fully characterize the molecular mechanism of reversing *NDC80^luti^* mediated repression.

In addition to its reversibility, *NDC80^luti^*-mediated repression can be adapted to fine-tune gene expression. Using a scalable expression system, we showed that modulating the levels of *NDC80^luti^* affects the efficiency of *NDC80^ORF^* repression (Figure 5). The higher the levels of *NDC80^luti^* transcription, the better the repression of *NDC80^ORF^* becomes. Notably, Set2 and Set3 are no longer required for repressing *NDC80^ORF^* when *NDC80^luti^* is highly expressed. One possible explanation is that the rate of nucleosome deposition at the *NDC80^ORF^* promoter is increased during higher levels of *NDC80^luti^* transcription. In this situation, the requirement for histone deacetylase complexes to stabilize nucleosomes becomes obsolete. Alternatively, elongating RNA polymerase might physically interfere with the *NDC80^ORF^* promoter when *NDC80^luti^* is highly expressed.

Whereas most studies have reported a binary switch for transcription interference mechanisms (Martens et al., 2004; Hongay et al., 2006; Camblong et al., 2007; Bumgarner et al., 2009; van Werven et al., 2012), we propose that transcriptional interference by expression of a 5’ extended transcript is tunable. This principle could be further adapted and used in synthetic genetic circuits to modulate gene expression levels. Indeed, mechanisms of transcriptional interference have been applied to coordinate activities of adjacent genes in both *E. coli* and budding yeast (Buetti-Dinh et al., 2009; Bordoy et al., 2016; Hao et al., 2016; Hoffmann et al., 2016).

### Concluding remarks

Transcriptional interference by 5’ extended isoforms might be wide-spread in yeast and across species. Transcript isoform sequencing in yeast revealed that the 5’ and 3’ ends of mRNAs are extremely heterogeneous (Pelechano et al., 2013). During budding yeast meiosis, more than 190 genes express an extended 5’ leader sequence (Brar et al., 2012). Some examples have been further confirmed and show a clear inhibitory effect on expression from the downstream promoter (Liu et al., 2015; Xie et al., 2016). However, how the expression of different mRNA isoforms regulate gene expression remains unexplored at the genome-wide level. In higher eukaryotes including human cells, a wide range of 5’ extended mRNA isoforms are also expressed often in a cell type-specific manner (Wang et al., 2008; Aanes et al., 2013; Brown et al., 2014). Understanding the principles underlying gene regulation by 5’ extended mRNA isoforms during yeast meiosis will deepen our understanding of how complex differentiation programs in higher eukaryotes are regulated.

## Materials and methods

### Yeast strains

Yeast strains used in this paper were derived from the sporulation proficient SK1 strain background, except for the strains that harbored the LexA/*lexO* system for which the W303 strain background was used. The genotypes are listed in Supplementary file 1. Gene or promoter deletions were generated using the one-step deletion protocol as described previously (Longtine et al., 1998). Tagging Sua7 with three copies of V5 epitope at C-terminus was performed using a one-step integration protocol using a V5-tagging cassette. The LexA/*lexO* system was described previously (Ottoz et al., 2014).

### Growth and conditions

The synchronous meiosis procedure using *pCUP-IME1* and *pCUP-IME4* was described, previously (Berchowitz et al., 2013; Chia and van Werven, 2016). In short, cells were grown to saturation overnight in YPD (1.0% (w/v) yeast extract, 2.0% (w/v) peptone, 2.0% (w/v) glucose, and supplemented with tryptophan (9.6 mg/l), uracil (2.4 mg/l) and adenine (1.2 mg/l)). These cells were then shifted to pre-sporulation medium (BYTA) (1.0% (w/v) yeast extract, 2.0% (w/v) tryptone, 1.0% (w/v) potassium acetate, 50 mM potassium phthalate) where they were grown for 16–18 hr. Subsequently, cells were centrifuged, washed with sterile milliQ water, centrifuged again and suspended in sporulation medium (SPO) (0.3% (w/v) potassium acetate and 0.02% (w/v) raffinose]) at OD_600_ = 2.5. After two hours, copper (II) sulphate (50 µM) was added to induce *IME1* and *IME4* expression from the *CUP1* promoter and initiate meiosis synchronously. For the synchronous experiments described in Figure 3B–3E, we shifted cells directly from saturated YPD culture to SPO medium, and induced *pCUP-IME1*/*pCUP-IME4* after two hours in SPO (Chia and van Werven, 2016).

For meiotic time-courses not synchronized by *pCUP-IME1/pCUP-IME4* (Figure 1A, left panel), cells were grown and treated as described previously (Falk et al., 2010). In short, cells were grown to saturation overnight in YPD. These cells were then shifted to BYTA where they were grown for another 16–18 hr. Subsequently, cells were washed with sterile milliQ water, and transferred to SPO medium (0.3% (w/v) potassium acetate and 0.02% (w/v) raffinose)).

For the return to growth experiments, *ndt80∆* cells were left for six hours in SPO to arrest them in meiotic prophase. Cells were then transferred to an equivalent volume of pre-warmed YPD. All experiments were performed at 30°C in a shaker incubator at 300 rpm.

For Figure 2E–F, cells were grown for 6–8 hr in YPD at 30°C, diluted to an OD_600_ of 0.002, transferred to YP (1.0% (w/v) yeast extract and 2.0% (w/v) peptone)+2% raffinose+2% galactose (YP-RG) and grown for another 16–18 hr. The cells were diluted to an OD_600_ of 0.2, grown for another 2.5–3 hr, diluted back to OD 0.2 and induced to express *NDC80^luti^* by the addition of 1 μM β-estradiol. 25–30 OD_600_ units of cells were collected for ChIP analyses at 0 hr and at 3 hr after induction.

For the time courses in Figure 3H, Figure 3—figure supplement 1H and I, cells were grown for 6–8 hr in YPD at 30°C, diluted to an OD_600_ of 0.002, transferred to YP-RG and grown for another 16–18 hr. Cells were diluted to an OD_600_ of 0.2 in YP-RG and *NDC80^luti^* expression was induced by the addition of 1 μM β-estradiol. Samples were taken at 0, 3, 4.5, and 6 hr after β-estradiol addition.

For Figure 4—figure supplement 1B, cells were grown to saturation overnight in YPD. These cells were then shifted to BYTA, in which they were grown for another 16–18 hr. Subsequently, cells were washed with sterile milliQ water, and transferred to SPO medium. After two hours in SPO, copper (II) sulphate (50 µM) was added to induce *IME1* and *IME4* from the *CUP1* promoter. At four hours in SPO, cells were either treated with ethanol or cycloheximide (0.2 mg/ml). Samples for western blotting were taken at 15, 30, 45, 60 and 90 min after adding ethanol or cycloheximide.

For Figure 5—figure supplement 1A, cells were grown for 16–18 hr in YPD at 30°C and then diluted to an OD_600_ of 0.2. Subsequently, the cells were grown for 2.5–3 hr to reach exponential phase. The cells were diluted again to an OD_600_ of 0.2 and induced to express *NDC80^luti^* with either 10 or 20 nM of β-estradiol. Cells were collected for qPCR analysis at 0 hr and at 3 hr after induction.

For Figure 5—figure supplement 1B and C, cells were grown in YPD at 30 ˚C overnight to saturation, diluted to OD_600_ of 0.1, and then grown to OD_600_ of 0.3–0.5 at 30 °C. Three OD_600 _units of cells were taken as the pre-induction samples. Cells were then diluted to an OD_600_ of 0.1 in YPD and split into three flasks. Subsequently, β–estradiol was added to the cells with a final concentration of either 15 nM or 25 nM. Ethanol was added as to the uninduced cells. 2 hr after β–estradiol induction,~3 OD_600 _units of cells were taken for western analysis, and at 4 hr, another ~3 OD_600 _units of cells were collected. All the samples were processed according to the western blotting protocol. The OD_600_ of each culture was also measured when the samples were taken. Equal OD_600_ units of samples was loaded during the gel electrophoresis.

### Nuclei/DAPI counting

DAPI staining was used to monitor meiotic divisions throughout meiotic time courses. Cells were fixed in 80% (v/v) ethanol, pelleted by centrifugation and re-suspended in PBS with DAPI (1 µg/ml). Cells were sonicated for a few seconds and left in the dark at room temperature for at least 5 min. The proportion of cells containing one, two, three, or four DAPI masses was counted using a fluorescence microscope. At least two independent biological experiments were performed for each meiotic time-course experiment.

### Flow cytometry analysis

Flow cytometry was used to monitor meiotic DNA replication as described previously (Chia and van Werven, 2016). Cells were fixed in 80% (v/v) ethanol and re-suspended in 50 mM Tris-HCl pH 7.5. Cells were sonicated for a few seconds and were treated with 0.2 mg/ml ribonuclease A in 50 mM Tris-HCl pH 7.5 at 37°C overnight. Cells were stained with 50 µg/ml propidium iodide in FACS buffer (200 mM Tris-HCl pH 7.5, 211 mM NaCl and 78 mM MgCl_2_) for one hour at room temperature before flow cytometry analysis (BD LSRFortessa, BD Biosciences). Propidium iodide stained cells were excited with a 561 nm yellow-green laser and signals were detected using a 610/20 yellow filter. Pulse shape analysis (pulse height against pulse area) was used to exclude clumps and doublets. DNA content from single cells was estimated with a histogram of counts against pulse area. At least 50,000 cells were used for the analysis.

### Western blotting

Western blotting was used to determine Ndc80 protein levels. Protein extracts were prepared by using trichloroacetic acid (TCA) extraction protocol. In short, cells were pelleted by centrifugation (~2400 g, 1 min, room temperature) and re-suspended in 5% w/v TCA for at least 10 min. For vegetative samples, pellets were washed with TE50 buffer (50 mM Tris pH 7.5, 1 mM EDTA), then with acetone, and completely dried. For meiotic samples, the pellets were only washed with acetone and dried. Furthermore, pellets were mixed with lysis buffer (50 mM Tris pH 7.5, 1 mM EDTA, 2.75 mM dithiothreitol (DTT)) and cells were disrupted using a mini beadbeater (BioSpec). Lysates were mixed with SDS loading buffer (187.5 mM Tris pH 6.8, 6.0 % v/v β-mercaptoethanol, 30% v/v Glycerol, 9.0 % w/v SDS, 0.05% w/v Bromophenol Blue) and boiled for 5 min for denaturation. After polyacrylamide gel electrophoresis, proteins were transferred onto PVDF or nitrocellulose membranes using the Mini Trans-Blot Cell (Bio-Rad (CA, USA)). The membranes were blocked for 60 min in blocking buffer (1% w/v BSA, 1% w/v milk) before incubation with mouse anti-V5 (R96025, Sigma-Aldrich (MO, USA)) at a 1:2000 dilution overnight at 4°C. Hxk1 loading control was detected using a rabbit anti-hexokinase antibody (H2035, Stratech (Newmarket, UK)) at a 1:8000 dilution overnight at 4°C. Membranes were then washed in PBST (phosphate buffered saline with 0.01% (v/v) Tween-20) and incubated with IRDye 800CW goat anti-mouse and IRDye 680RD donkey anti-rabbit secondary antibodies (LI-COR (NE, USA)) at a 1:15000 dilution. Protein levels were detected using an Odyssey Imager (LI-COR). To detect HA tagged Ime1, a mouse anti-HA antibody was used at a 1:2000 dilution (901501, Biolegend (CA, USA)). All other steps were performed as above. At least two independent biological experiments were performed for each western blot experiment.

To measure bulk histone H3 levels, membranes were blocked for 60 min in blocking buffer (5% w/v milk) before incubation with rabbit anti-H3, C terminus (07–690, Millipore (MA, USA)) at a 1:3000 dilution overnight at 4°C. To measure bulk H3K36me3 levels, membranes were blocked for 60 min in blocking buffer (1% w/v BSA, 1% w/v milk) before incubation with rabbit anti-H3K36me3 (Ab9050, Abcam (Cambridge, UK)) at a 1:1000 dilution overnight at 4°C. Membranes were then washed in PBST and incubated with HRP conjugated ECL donkey anti-rabbit secondary antibodies (GE Healthcare (IL, USA)) at a 1:8000 dilution. After addition of ECL substrate (GE Healthcare), protein levels were detected using an ImageQuant RGB 600 machine (GE Healthcare). At least two independent biological experiments were performed for each western blot experiment.

### RT-qPCR

To quantify *NDC80^luti^* mRNA levels as described in Figure 4—figure supplement 1A, we used a reverse transcription combined with quantitative PCR (RT-qPCR) protocol. Total RNA was isolated, purified and treated with DNAse (Macherey*-*Nagel (Düren, Germany)). 750 ng of total RNA was reverse-transcribed using random primers and Protoscript II (NEB (MA, USA)), and single-stranded cDNA was quantified by real-time PCR using SYBR green mix (Life Technologies). The signals were normalized to *ACT1* transcript levels. The oligonucleotide sequences used for RT-PCR experiments are displayed in Supplementary file 2.

For the RT-qPCR in Figure 5—figure supplement 1A, RNA was isolated by acid phenol-chloroform extraction, treated with DNase (TURBO DNA-free kit, Thermo Fisher (MA, USA)), and reverse transcribed into cDNA (Superscript III Supermix, Thermo Fisher). The cDNA was quantified using the Absolute Blue qPCR Mix (Thermo Fisher). The *NDC80^luti^* signals were normalized to *ACT1* transcript levels. The oligonucleotide sequences used for RT-qPCR experiments are displayed in Supplementary file 2.

### Chromatin immunoprecipiation

Chromatin immunoprecipitation (ChIP) experiments were performed as described previously. Cells were fixed in 1.0% v/v of formaldehyde for 15–20 min at room temperature and quenched with 100 mM glycine. Cells were broken using a mini beadbeater (BioSpec) and crosslinked chromatin was sheared by sonication using a Bioruptor (Diagenode (Seraing, Belgium), 7 cycles of 30 s on/off). Extracts were incubated for 2 hr or overnight at 4 ˚C with magnetic Prot A beads (Sigma) coupled with a polyclonal antibody against Histone H3 trimethyl lysine 36 (Ab9050, Abcam), Histone H3 dimethyl lysine 4 (Ab32356, Abcam) or Histone H3 (Ab1791, Abcam). Subsequently, reverse cross-linking was done in Tris-EDTA buffer (100 mM Tris pH 8.0, 10 mM EDTA, 1.0% v/v SDS) at 65°C overnight. After 2 hr of proteinase K treatment, samples were cleaned up and H3K36me3 enrichment was measured by real-time PCR using SYBR green mix (Life Technologies (CA, USA)) and primers corresponding to the *NDC80* promoter and the 5’ region of the *NDC80* open reading frame. Sua7-V5 binding was measured using similar procedures, except that anti-V5 agarose beads (Sigma-Aldrich) were used instead. The oligonucleotide sequences used for ChIP experiments are displayed in Supplementary file 2.

### ChIP on micrococcal nuclease (MNase) treated chromatin extracts

To determine the chromatin structure at the *NDC80* locus, we extracted mononucleosomes using a MNase digestion protocol that was described previously followed by ChIP for histone H3 (Rando, 2010, 2011). Approximately 250 OD_600_ units of cells were crosslinked for 15 min with formaldehyde (1% v/v) and the reaction was quenched with glycine (125 mM). Subsequently, cells were resuspended in 20 ml of buffer Z (1 M sorbitol, 50 mM Tris-HCl pH 7.4) plus β-mercaptoethanol (10 mM) and treated with 250 μg of T100 Zymolase (MP Biomedicals (CA, USA)) for 60 min. Next, cells were resuspended in 2.5 ml NP buffer (0.5 mM spermidine, 1 mM β-mercaptoethanol (β -ME), 0.075% (w/v) Tergitol solution-type NP-40 detergent (NP-40), 50 mM NaCl, 10 mM Tris-HCl pH 7.4, 5 mM MgCl_2_, 1 mM CaCl_2_), and extract was treated with 5, 0.625, 0.2 or 0.04 μl of MNase (2 mg/ml, NEB) for 30 min at 37°C, the reaction was quenched with EDTA (10 mM). The extract was adjusted to 0.1 M HEPES-KOH pH 7.5, 150 mM NaCl, 0.1% w/v sodium deoxycholate, and 1% w/v Triton X-100. To check for the extent of MNase digestion, 60 μl of MNase treated and untreated extracts were reverse crosslinked overnight in SDS-TE (1% (w/v) SDS, 10 mM Tris pH 8, 1 mM EDTA), treated with RNase A and purified DNA fragments were separated by gel electrophoresis. The extracts which the showed a mono-nucleosome pattern were used for ChIP with histone H3 antibodies. The ChIP was performed with 600 μl of extract as described in the chromatin immunoprecipitation section of the Materials and methods. ChIP samples were quantified by qPCR on a 7500 FAST Real-Time PCR machine (Applied Biosystems (CA, USA)). Scanning primer pairs covering the *NDC80* locus and upstream region were used for the analysis. Signals were quantified relative to untreated genomic DNA, and normalized over a primer pair directed against the *PHO5* core promoter (Chang and Vancura, 2012). The oligonucleotide sequences are available in Supplementary file 2.

### Northern blotting

We adapted a northern blot protocol that was described previously (Koster et al., 2014). In short, RNA was extracted with Acid Phenol:chloroform:IAA (125:24:1) and precipitated in ethanol with 0.3M sodium acetate. RNA samples were denatured in a glyoxal/DMSO mix (1M deionized glyoxal, 50% v/v DMSO, 10 mM sodium phosphate (NaPi) buffer pH 6.5–6.8) at 70°C for 10 min. Denatured samples were mixed with loading buffer (10% v/v glycerol, 2 mM NaPi buffer pH 6.5–6.8, 0.4% w/v bromophenol blue) and separated on an agarose gel (1.1% w/v agarose, 0.01M NaPi buffer) for at least 3 hr at 80 V. RNAs were then transferred onto nylon membranes overnight by capillary transfer. rRNA bands were visualized by methylene blue staining. The membranes were blocked for at least 3 hr at 42°C in ULTRAhyb Ultrasensitive Hybridization Buffer (Thermo Fisher) before hybridization. The radioactive probes specific to *NDC80, SCR1* and *CIT1* were synthesized using a Prime-It II Random Primer Labeling Kit (Agilent (CA, USA)), a DNA template and ATP (α−32P) (Perkin-Elmer (MA, USA)). The oligo-nucleotide sequences used for amplifying the *NDC80, CIT1* or *SCR1* templates are displayed in Supplementary file 2. At least two independent biological experiments were performed for each northern blot experiment.

### Quantification of northern and western blots

*NDC80^ORF^, NDC80^luti^, CIT1* and *SCR1* levels were estimated from northern blots using ImageJ (Schneider et al., 2012). The net intensity of each band of interest was determined by subtracting the mean background intensity of the areas immediately above and below the band. Signals were first normalized to *SCR1* levels and further normalized to a specific band on the same membrane (usually the first time point when either *NDC80^ORF^* or *NDC80^luti^* appeared) For Figure 3E, one-tailed, unpaired t tests were conducted to determine if the difference in *NDC80^ORF^* levels between mutant and control strains were statistically significant.

Intensities of Ndc80 and Hxk1 bands on western blots were quantified using Image Studio Lite (LI-COR). Ndc80 levels were first normalized to Hxk1 levels and further normalized to that of the first time point on the same membrane.

### Spot growth assay

For strains harboring *NDC80^luti^* under control of the *GAL1-10* promoter, cells were first grown on YP plus 2% glycerol (YPG) plates overnight, and then re-suspended in milliQ H_2_O to an OD_600_ of 0.2. Next, 5-fold serial dilutions were performed and diluted cells were spotted onto either YP-RG plates with no β-estradiol or YP-RG plates supplemented with1 μM β-estradiol. The cells were incubated at 30°C for 1–2 days. Note that the *GAL1-10* promoter in the SK1 strain background does not directly respond to galactose. At least two independent biological experiments were performed for each spot assay experiment.

For strains harboring constructs in which *NDC80^luti^* expression is driven by LexA/*lexO*, cells were grown on YPD plates, re-suspended in milliQ water to an OD_600_ of 0.2, serially diluted as above, and then spotted onto either YPD plates with no β-estradiol or YPD plates with different concentrations of β-estradiol (10, 15, 20, 25, or 30 nM). The cells were incubated at 30°C for 1 day before imaging. At least two independent biological experiments were performed for each spot assay experiment.

## Additional files

Supplementary file 1. Table of yeast strains used in this study. Table describing genotypes of the strains.

Supplementary file 2. Table of oligo nucleotide sequences used in this study. Table describing oligo nucleotide sequences used for quantitative PCR and for preparing the northern blot probes.

Transparent reporting form
