# Supplementary material for: Transcription of a 5’ extended mRNA isoform directs dynamic chromatin changes and interference of a downstream promoter
Source: eLife. 2017 Sep 14;6:e27420. doi: 10.7554/eLife.27420 (PMC5655139; doi:10.7554/eLife.27420)
Supplement: Supplementary file 1. — Table describing genotypes of the strains. [file elife-27420-supp1.docx]

**Supplementary File 1. Table of yeast strains used in this study**

| **Strain** | **Genotype** |
| --- | --- |
| *FW1472* | *MAT****a****, ho::LYS2, lys2, ura3, leu2::hisG, his3::hisG, trp1::hisG, irt1::pCUP-3HA-IME1::KanMX, ime4::pCUP-3HA-IME4::KanMX, NDC80-3V5::KanMX, set2::His3MX*  *MATα, ho::LYS2, lys2, ura3, leu2::hisG, his3::hisG, trp1::hisG, irt1::pCUP-3HA-IME1:: KanMX, ime4::pCUP-3HA-IME4:: KanMX, NDC80-3V5::KanMX, set2::His3MX* |
| *FW1509* | *MAT****a****, ho::LYS2, lys2, ura3, leu2::hisG, his3::hisG, trp1::hisG* |
| *FW1510* | *MATα, ho::LYS2, lys2, ura3, leu2::hisG, his3::hisG, trp1::hisG* |
| *FW1511* | *MAT****a****, ho::LYS2, lys2, ura3, leu2::hisG, his3::hisG, trp1::hisG*  *MATα, ho::LYS2, lys2, ura3, leu2::hisG, his3::hisG, trp1::hisG* |
| *FW1868* | *MAT****a****, ho::LYS2, lys2, ura3, leu2::hisG, his3::hisG, trp1::hisG, irt1::pCUP-3HA-IME1::NatMX, ime4::pCUP-3HA-IME4::NatMX, NDC80-3V5::KanMX, NDC80::pndc80(600-300)::His3MX*  *MATα, ho::LYS2, lys2, ura3, leu2::hisG, his3::hisG, trp1::hisG, irt1::pCUP-3HA-IME1:: NatMX, ime4::pCUP-3HA-IME4:: NatMX, NDC80-3V5::KanMX, NDC80::pndc80(600-300)::His3MX* |
| *FW1902* | *MAT****a****, ho::LYS2, lys2, ura3, leu2::hisG, his3::hisG, trp1::hisG, irt1::pCUP-3HA-IME1::HphMX, ime4::pCUP-3HA-IME4::NatMX, NDC80-3V5::KanMX*  *MATα, ho::LYS2, lys2, ura3, leu2::hisG, his3::hisG, trp1::hisG, irt1::pCUP-3HA-IME1:: HphMX, ime4::pCUP-3HA-IME4:: NatMX, NDC80-3V5::KanMX* |
| *FW1922* | *MAT****a****, ho::LYS2, lys2, ura3, leu2::hisG, his3::hisG, trp1::hisG, irt1::pCUP-3HA-IME1::HphMX, ime4::pCUP-3HA-IME4::NatMX, NDC80-3V5::KanMX, set2::His3MX, set3::His3MX*  *MATα, ho::LYS2, lys2, ura3, leu2::hisG, his3::hisG, trp1::hisG, irt1::pCUP-3HA-IME1:: HphMX, ime4::pCUP-3HA-IME4:: NatMX, NDC80-3V5::KanMX, set2::His3MX, set3::His3MX* |
| *FW2928* | *MAT****a****, ho::LYS2, lys2, ura3, leu2::hisG, his3::hisG, trp1::hisG, irt1::pCUP-3HA-IME1::HphMX, ime4::pCUP-3HA-IME4::NatMX, NDC80-3V5::KanMX, set3::His3MX*  *MATα, ho::LYS2, lys2, ura3, leu2::hisG, his3::hisG, trp1::hisG, irt1::pCUP-3HA-IME1:: HphMX, ime4::pCUP-3HA-IME4:: NatMX, NDC80-3V5::KanMX, set3::His3MX* |
| *FW2929* | *MAT****a****, ho::LYS2, lys2, ura3, leu2::hisG, his3::hisG, trp1::hisG, irt1::pCUP-3HA-IME1::HphMX, ime4::pCUP-3HA-IME4::NatMX, NDC80-3V5::KanMX, set2::His3MX*  *MATα, ho::LYS2, lys2, ura3, leu2::hisG, his3::hisG, trp1::hisG, irt1::pCUP-3HA-IME1:: HphMX, ime4::pCUP-3HA-IME4:: NatMX, NDC80-3V5::KanMX, set2::His3MX* |
| *FW2957* | *MAT****a****, ho::LYS2, lys2, ura3, leu2::hisG, his3::hisG, trp1::hisG, irt1::pCUP-3HA-IME1::HphMX, ime4::pCUP-3HA-IME4::NatMX, SUA7-3V5::KanMX*  *MATα, ho::LYS2, lys2, ura3, leu2::hisG, his3::hisG, trp1::hisG, irt1::pCUP-3HA-IME1:: HphMX, ime4::pCUP-3HA-IME4:: NatMX, SUA7-3V5::KanMX* |
| *FW3033* | *MAT****a****, ho::LYS2, lys2, ura3, leu2::hisG, his3::hisG, trp1::hisG, irt1::pCUP-3HA-IME1::HphMX, ime4::pCUP-3HA-IME4::NatMX, set1::KanMX*  *MATα, ho::LYS2, lys2, ura3, leu2::hisG, his3::hisG, trp1::hisG, irt1::pCUP-3HA-IME1:: HphMX, ime4::pCUP-3HA-IME4:: NatMX, set1::KanMX* |
| *FW3856* | *MAT****a****, ho::LYS2, lys2, ura3, leu2::hisG, his3::hisG, trp1::hisG, irt1::pCUP-3HA-IME1::HphMX, ime4::pCUP-3HA-IME4::NatMX, NDC80-3V5::KanMX, ndt80::LEU2*  *MATα, ho::LYS2, lys2, ura3, leu2::hisG, his3::hisG, trp1::hisG, irt1::pCUP-3HA-IME1:: HphMX, ime4::pCUP-3HA-IME4:: NatMX, NDC80-3V5::KanMX, ndt80::LEU2* |
| *FW4644* | *MAT****a****, ho::LYS2, lys2, ura3, leu2::hisG, his3::hisG, trp1::hisG, NDC80-3V5::KanMX*  *MATα, ho::LYS2, lys2, ura3, leu2::hisG, his3::hisG, trp1::hisG, NDC80-3V5::KanMX* |
| *FW5530* | *MAT****a****, ho::LYS2, lys2, ura3, leu2::hisG, his3::hisG, trp1::hisG, SUA7-3V5::Kanmx, irt1::pCUP-3HA-IME1:: HphMX, ime4::pCUP-3HA-IME4:: NatMX, NDC80::pndc80(600-300)::His3MX*  *MATα, ho::LYS2, lys2, ura3, leu2::hisG, his3::hisG, trp1::hisG, SUA7-3V5::Kanmx, irt1::pCUP-3HA-IME1:: HphMX, ime4::pCUP-3HA-IME4:: NatMX, NDC80::pndc80(600-300)::His3MX* |
| *UB91* | *MAT****a****, ho::LYS2, lys2, ura3, leu2::hisG, his3::hisG, trp1::hisG, ura3::pGPD1-GAL4(848).ER::URA3* |
| *UB1217* | *MAT****a****, ho::LYS2, lys2, ura3, leu2::hisG, his3::hisG, trp1::hisG, HISMX:pGAL-Ndc80-3V5:KanMX, ura3::pGPD1-GAL4(848).ER::URA3*  *(pGAL integrated 536 bp upstream of Ndc80 AUG)* |
| *UB1218* | *MATalpha, ho::LYS2, lys2, ura3, leu2::hisG, his3::hisG, trp1::hisG, HISMX:pGAL-Ndc80-3V5:KanMX, ura3::pGPD1-GAL4(848).ER::URA3*  *(pGAL integrated 536 bp upstream of Ndc80 AUG)* |
| *UB1235* | *MATα, ho::LYS2, ura3, leu2::hisG, his3::hisG, trp1::hisG, set2::hismx, set3::his3mx, HISMX:pGAL-Ndc80-3V5:KanMX, ura3::pGPD1-GAL4(848).ER::URA3*  *(pGAL integrated 536 bp upstream of Ndc80 AUG)* |
| *UB1236* | *MATα, ho::LYS2, ura3, leu2::hisG, his3::hisG, trp1::hisG, set2::hismx, HISMX:pGAL-Ndc80-3V5:KanMX, ura3::pGPD1-GAL4(848).ER::URA3*  *(pGAL integrated 536 bp upstream of Ndc80 AUG)* |
| *UB1237* | *MATα, ho::LYS2, ura3, leu2::hisG, his3::hisG, trp1::hisG, set3::his3mx, HISMX:pGAL-Ndc80-3V5:KanMX, ura3::pGPD1-GAL4(848).ER::URA3*  *(pGAL integrated 536 bp upstream of Ndc80 AUG)* |
| *UB1240* | *MAT****a****, ho::LYS2, lys2, ura3, leu2::hisG, his3::hisG, trp1::hisG, Ndc80-3V5:KanMX, ura3::pGPD1-GAL4(848).ER::URA3* |
| *UB1252* | *MATα, ho::LYS2, lys2, ura3, leu2::hisG, his3::hisG, trp1::hisG, Ndc80-3V5:KanMX, ura3::pGPD1-GAL4(848).ER::URA3* |
| *UB3338* | *MAT****a****, ho::LYS2, lys2, ura3, leu2::hisG, his3::hisG, trp1::hisG, ura3::pGPD1-GAL4(848).ER::URA3, HISMX:pGAL-Ndc80*  *(pGAL integrated 536 bp upstream of Ndc80 AUG)* |
| *UB3351* | *MATα, ho::LYS2, lys2, ura3, leu2::hisG, his3::hisG, trp1::hisG, ura3::pGPD1-GAL4(848).ER::URA3, ndc80∆:KanMX4, leu2::NDC80-3V5:LEU2* |
| *UB3370* | *MATα, ho::LYS2, lys2, ura3, leu2::hisG, his3::hisG, trp1::hisG, GAL-NDT80::TRP1, ura3::pGPD1-GAL4(848).ER::URA3, ndc80∆:KanMX4, leu2::NDC80-3V5:LEU2* |
| *UB3545* | *MATα, ho::LYS2, lys2, ura3, leu2::hisG, his3::hisG, trp1::hisG, set2::his3mx, Ndc80-3V5:KanMX, ura3::pGPD1-GAL4(848).ER::URA3* |
| *UB3547* | *MATα, ho::LYS2, lys2, ura3, leu2::hisG, his3::hisG, trp1::hisG, set3::his3mx, Ndc80-3V5:KanMX, ura3::pGPD1-GAL4(848).ER::URA3* |
| *UB3549* | *MATα, ho::LYS2, lys2, ura3, leu2::hisG, his3::hisG, trp1::hisG, set2::hismx, set3::his3mx, Ndc80-3V5:KanMX, ura3::pGPD1-GAL4(848).ER::URA3* |
| *UB5154* | *MATα, ho::LYS2, lys2, ura3, leu2::hisG, his3::hisG, trp1::hisG, ura3::pGPD1-GAL4(848).ER::URA3, ndc80∆:KanMX4, leu2::pGAL-NDC80-3V5:LEU2*  *(pGAL integrated 536 bp upstream of Ndc80 AUG)* |
| *UB8110* | *MAT****a****, ho::LYS2, lys2, ura3, leu2::hisG, his3::hisG, trp1::hisG, ura3::pGPD1-GAL4(848).ER::URA3, Ndc80-3V5:KanMX, set2::HygB, set3::CNAT* |
| *UB8114* | *MATa, ho::LYS2, lys2, ura3, leu2::hisG, his3::hisG, trp1::hisG, ura3::pGPD1-GAL4(848).ER::URA3, HISMX:pGAL-Ndc80-3V5:KanMX, set2::HygB, set3::CNAT*  *(pGAL integrated 536 bp upstream of Ndc80 AUG)* |
| *UB8358* | *MAT****a****, ADE2, leu2-3, ura3, trp1-1, his3-11,15, can1-100, GAL, phi+, KanMX:p1X-LexO-pCyc1-Ndc80luti, trp1::pGPD1-LexA-ER-HA-B112::TRP1*  *W303*  *(1X-LexO-pCyc1 integrated 536 bp upstream of Ndc80 AUG, thus replacing the Ndc80luti promoter)* |
| *UB8362* | *MAT****a****, ADE2, leu2-3, ura3, trp1-1, his3-11,15, can1-100, GAL, phi+, KanMX:p2X-LexO-pCyc1-Ndc80luti, trp1::pGPD1-LexA-ER-HA-B112::TRP1*  *W303*  *(2X-LexO-pCyc1 integrated 536 bp upstream of Ndc80 AUG, thus replacing the Ndc80luti promoter)* |
| *UB8366* | *MAT****a****, ADE2, leu2-3, ura3, trp1-1, his3-11,15, can1-100, GAL, phi+, KanMX:p3X-LexO-pCyc1-Ndc80luti, trp1::pGPD1-LexA-ER-HA-B112::TRP1*  *W303*  *(3X-LexO-pCyc1 integrated 536 bp upstream of Ndc80 AUG, thus replacing the Ndc80luti promoter)* |
| *UB8370* | *MAT****a****, ADE2, leu2-3, ura3, trp1-1, his3-11,15, can1-100, GAL, phi+, KanMX:p8X-LexO-pCyc1-Ndc80luti, trp1::pGPD1-LexA-ER-HA-B112::TRP1*  *W303*  *(8X-LexO-pCyc1 integrated 536 bp upstream of Ndc80 AUG, thus replacing the Ndc80luti promoter)* |
| *UB8374* | *MAT****a****, ADE2, leu2-3, ura3, trp1-1, his3-11,15, can1-100, GAL, phi+, trp1::pGPD1-LexA-ER-HA-B112::TRP1*  *W303* |
| *UB8686* | *MAT****a****, ADE2, leu2-3, ura3, trp1-1, his3-11,15, can1-100, GAL, psi+, KanMX:p3X-LexO-pCyc1-Ndc80luti, trp1::pGPD1-LexA-ER-HA-B112::TRP1, set2::HygB, set3::CNAT*  *W303*  *(3X-LexO-pCyc1 integrated 536 bp upstream of Ndc80 AUG, thus replacing the Ndc80luti promoter)* |
| *UB8691* | *MAT****a****, ADE2, leu2-3, ura3, trp1-1, his3-11,15, can1-100, GAL, psi+, trp1::pGPD1-LexA-ER-HA-B112::TRP1, set2::HygB, set3::CNAT*  *W303* |
| *UB8693* | *MAT****a****, ADE2, leu2-3, ura3, trp1-1, his3-11,15, can1-100, GAL, phi+, KanMX:p8X-LexO-pCyc1-Ndc80luti, trp1::pGPD1-LexA-ER-HA-B112::TRP1, set2::HygB, set3::CNAT*  *W303*  *(8X-LexO-pCyc1 integrated 536 bp upstream of Ndc80 AUG, thus replacing the Ndc80luti promoter)* |
| *UB9181* | *MATα, ho::LYS2, lys2, ura3, leu2::hisG, his3::hisG, trp1::hisG, ura3::pGPD1-GAL4(848).ER::URA3, ndc80∆:KanMX4, leu2::pGAL-NDC80-3V5:LEU2, pGAL-NDT80::TRP1*  *(pGAL integrated 536 bp upstream of NDC80 AUG)* |
| *UB9921* | *MATα, ho::LYS2, lys2, ura3, leu2::hisG, his3::hisG, trp1::hisG, GAL-NDT80::TRP1, ura3::pGPD1-GAL4(848).ER::URA3, leu2::pGAL-mse-NDC80-3V5:LEU2, ndc80∆:KanMX4*  *(pGAL integrated 536 bp upstream of NDC80 AUG)* |
| *UB9923* | *MATα, ho::LYS2, lys2, ura3, leu2::hisG, his3::hisG, trp1::hisG, ura3::pGPD1-GAL4(848).ER::URA3, leu2::pGAL-mse-NDC80-3V5:LEU2, ndc80∆:KanMX4*  *(pGAL integrated 536 bp upstream of NDC80 AUG)* |
| *UB12945* | *MATa, ADE2, leu2-3, ura3, trp1-1, his3-11,15, can1-100, GAL, phi+, NDC80-3V5:HisMX, trp1::pGPD1-LexA-ER-HA-B112::TRP1*  W303 |
| *UB12947* | *MATa, ADE2, leu2-3, ura3, trp1-1, his3-11,15, can1-100, GAL, phi+, NDC80-3V5:HisMX, trp1::pGPD1-LexA-ER-HA-B112::TRP1, set2::HygB, set3::CNAT*  W303 |
| *UB12949* | *MATa, ADE2, leu2-3, ura3, trp1-1, his3-11,15, can1-100, GAL, phi+, KanMX:p8X-LexO-pCyc1-Ndc80luti-NDC80-3V5:HisMX, trp1::pGPD1-LexA-ER-HA-B112::TRP1*  W303  **8X-LexO-pCyc1 integrated 536 bp upstream of Ndc80 AUG, thus replacing the Ndc80luti promoter** |
| *UB12951* | *MATa, ADE2, leu2-3, ura3, trp1-1, his3-11,15, can1-100, GAL, phi+, KanMX:p8X-LexO-pCyc1-Ndc80luti-NDC80-3V5:HisMX, trp1::pGPD1-LexA-ER-HA-B112::TRP1, set2::HygB, set3::CNAT*  W303  **8X-LexO-pCyc1 integrated 536 bp upstream of Ndc80 AUG, thus replacing the Ndc80luti promoter** |
| *UB1026* | *MAT****a****, ho::LYS2, lys2, ura3, leu2::hisG, his3::hisG, trp1::hisG, ime1::pCUP-3HA-IME1::KanMX*  *MATα, ho::LYS2, lys2, ura3, leu2::hisG, his3::hisG, trp1::hisG, ime1::pCUP-3HA-IME1::KanMX* |
