# Supplementary material for: Transcription of a 5’ extended mRNA isoform directs dynamic chromatin changes and interference of a downstream promoter
Source: eLife. 2017 Sep 14;6:e27420. doi: 10.7554/eLife.27420 (PMC5655139; doi:10.7554/eLife.27420)
Supplement: Supplementary file 2. — Table describing oligo nucleotide sequences used for quantitative PCR and for preparing the northern blot probes. [file elife-27420-supp2.docx]

**Supplementary File 2. Table of oligo nucleotide sequences used in this study**

| **Primer name** | **Oligo nucleotide sequence 5’ to 3’** |
| --- | --- |
| *ACT1_FW* | gtaccaccatgttcccaggtatt |
| *ACT1_RV* | agatggaccactttcgtcgt |
| *HMR_FW* | ACGATCCCCGTCCAAGTTATG |
| *HMR_RV* | CTTCAAAGGAGTCTTAATTTCCCTG |
| *NDC80probe_FW* | GGAGAGGTAGAATCGTCCCTG |
| *NDC80probe_RV* | CTCCTCTTGAATAGCGCTTTGG |
| *NDC80_1_FW* | GCTCCTGTGTTCTCCATT |
| *NDC80_1_RV* | GTGTGTTGATACTGCACTG |
| *NDC80_2_FW* | ACCCGGATATCTGTTCAGCC |
| *NDC80_2_RV* | TGTGGCGAATTGTTGCTCTT |
| *NDC80_3_FW* | CGCCACAAGAAGGTCTC |
| *NDC80_3_RV* | GCTTTTCGGACCTCCAAC |
| *NDC80_4_FW* | GTTGGAGGTCCGAAAAGC |
| *NDC80_4_RV* | GTTCAGTTATAACCATCTGGCAC |
| *NDC80_5_FW* | GTGCCAGATGGTTATAACTGAAC |
| *NDC80_5_RV* | CCGCTAATCGCAATAGACTG |
| *NDC80_6_FW* | GGTTGAGAGCCCCGTTAAGT |
| *NDC80_6_RV* | TTGGCACTTTCAGTATGGGT |
| *NDC80_7_FW* | CCCATACTGAAAGTGCCAAAAGA |
| *NDC80_7_RV* | GGGACGATTCTACCTCTCCTGTG |
| *NDC80_8_FW* | GGAATACATTCACAGGAGAGG |
| *NDC80_8_RV* | GGAATATATTATAGTACACCCTAACG |
| *NDC80_9_FW* | TGCAAAGCTCAACAAGTACTGA |
| *NDC80_9_RV* | TGCAGTTGGTATTTGGGACG |
| *NDC80_10_FW* | CAAGGTCTAACCGACATGATC |
| *NDC80_10_RV* | CATTTGTACCTCCTGCAAC |
| *PHO5-TATA_FW* | CCATTTGGGATAAGGGTAAACATC |
| *PHO5-TATA_RV* | AGAGATGAAGCCATACTAACCTCG |
| *SCR1_probe_F* | GAAGTGTCCCGGCTATAATAAA |
| *SCR1_probe_R* | GACGCTGGATAAAACTCCCC |
| *CIT1_probe_F* | CCGTGTTAGACCCCGAAGAAG |
| *CIT1_probe_R* | GGGCAGAAACGTTACCACCTTC |
