# Supplementary material for: Transcription of a 5’ extended mRNA isoform directs dynamic chromatin changes and interference of a downstream promoter
Source: eLife. 2017 Sep 14;6:e27420. doi: 10.7554/eLife.27420 (PMC5655139; doi:10.7554/eLife.27420)
Supplement: Peer review history

# Decision letter

**DOI:** 10.7554/eLife.27420.015

**Affiliations:** Howard Hughes Medical Institute, Memorial Sloan Kettering Cancer CenterUnited States

In the interests of transparency, eLife includes the editorial decision letter and accompanying author responses. A lightly edited version of the letter sent to the authors after peer review is shown, indicating the most substantive concerns; minor comments are not usually included.

Thank you for submitting your article "Transcription of a 5' extended mRNA isoform directs dynamic chromatin changes and interference of a downstream promoter" for consideration by *eLife*. Your article has been reviewed by three peer reviewers, and the evaluation has been overseen by a Reviewing Editor and Jessica Tyler as the Senior Editor. The reviewers have opted to remain anonymous.

The reviewers have discussed the reviews with one another and the Reviewing Editor has drafted this decision to help you prepare a revised submission. As you will see from the comments below, there was enthusiasm for this manuscript as a companion to the co-submitted manuscript from Chen et al. However, there were also several important weaknesses that need to be addressed before the paper can be published in *eLife*. We realize that we are requesting a substantial amount of work to strengthen the experiments and their conclusions, but given that your manuscript is a co-submission, we considered that it was fairer to give you the opportunity to revise the paper rather than to reject it and keep the door open to a resubmission at a later date.

Summary:

In this manuscript, the authors provide mechanistic insight into how an mRNA that initiates ~500bp upstream of the *NDC80* gene represses transcription from the NDC80ORF promoter. In the accompanying manuscript, basic features of the *NDC80* regulatory system were described, including demonstration that the long mRNA, termed NDC80luti, and the ORF mRNA appear anti-correlated and that blocking transcription from the NDC80luti promoter derepresses the ORF transcript. In this manuscript, the authors find support for a transcriptional interference mechanism in which transcription from the upstream start inhibits PIC assembly at the ORF promoter and establishes a histone modification pattern at the ORF promoter (enrichment of H3K4me2 and H3K36me3) that has been previously associated with recruitment of two well characterized yeast HDACs, Set3C and Rpd3S. The authors extend their work by showing that the *NDC80* regulatory system is reversible, when cells are switched from sporulation to growth media, and tunable, when the levels of the NDC80luti transcript are systematically elevated through a lexA-ER system.

Demonstration of the molecular basis of the repression mechanism is a valuable complement to the accompanying paper by Chen et al. However, as the authors acknowledge, involvement of cis-repression of a promoter by an overlapping non-coding transcript via transcription-coupled histone methylation and establishment of repressive chromatin is not a novel mechanism, being established at several other genes in yeast. In addition to the examples for this cited by the authors, there is the case of the upstream ncRNA that represses SER3 by promoting nucleosome assembly over the SER3 promoter, and antisense transcripts that mediate histone methylation of the promoter for the sense transcript at FLO11. Other studies, including recent work from the Buratowski lab on a genome-wide scale, showed that noncoding transcription across promoters leads to enrichment of H3K4me2 and H3K36me3 and repression of the promoters. With that said, the study does provide insight into an interesting example of regulated transcriptional interference.

In addition to the need for a more thorough coverage of relevant precedents for similar examples of gene regulation (see above), there are some important control experiments lacking, notably a failure to measure by ChIP the total H3 levels in the *NDC80^ORF^* promoter – to distinguish nucleosomes from non-histone chromatin protein occupancy, and to normalize changes in methylation for changes in H3 occupancy – and lack of quantification of changes in the *NDC80^ORF^* transcript, which is required to firmly establish the role of Set2/Set3 in repressing this promoter. In addition, there are a number of instances where direct experimental evidence is insufficient to support mechanistic claims made; these instances need to be remedied by inclusion of more data or by significant rewriting of text to soften conclusions and make them more nuanced. Statistical analysis of replicate data is missing for measurements of mRNA levels by northern analysis. Loading and normalization controls are lacking in multiple figures.

*Essential revisions:*

1) The increase in MNase-resistance in the *NDC80^ORF^* promoter region is not necessarily due to nucleosomes as it has been shown that non-histone protein binding in NDRs can also confer MNase-resistance (PMID: 28157509). For this reason, a ChIP of the MNase-resistant chromatin with histone antibodies is required to establish nucleosome versus non-histone protein occupancy.

2) In Figure 2C changes in nucleosome occupancy are inferred upon repression of the downstream promoter (at -100), but there is no evidence for loss of nucleosomes accompanying activation of the upstream promoter. Is this because the upstream promoter is nucleosome free even when repressed or is there another explanation?

3) Figure 2. Some information on the sequences of the promoters should be presented. Does the Sua7 occupancy profile make sense with respect to positions of core promoter elements? Why is the highest Sua7 signal detected 400bp upstream of the *NDC80^luti^* TSS?

4) Figure 2. How were the nucleosome positioning data normalized? Typically these data are normalized to a known, highly occupied nucleosome position (for example, Sekinger et al. Mol Cell 18: 735). Also control gels should be included to show the MNase digestion profile.

5) ChIP with histone antibodies is also required to normalize the H3-K36me3 and H3-K4me2 ChIP signals in Figure 3A-D and Figure 3—figure supplement 1 in order to establish that the amount of methylation per nucleosome is increased over the *NDC80^ORF^* promoter in a manner dependent on the *luti* promoter. K4me2 levels should be included in Figures 3C and 3D.

6) Figure 4A: RNA loading controls are needed in the northern blots. Quantification of the mRNA from replicates with a loading control is needed to establish that the *NCD80^ORF^* transcript is elevated reduced in prophase in the double mutant relative to WT, as this seems to be limited to the 3hr timepoint, and even this increase is not evident in Figure 4D. Perhaps the only meaningful increase in terms of Ndc80p expression occurs at 4-5hr.

7) Figure 4G. How many repetitions were done to calculate the relative Ndc80 levels? Also, the levels of the Hxk1 loading control seem to be changing both in this panel and in Figure 4—Figure supplement 1. This is perhaps not surprising given the change in carbon source in this experiment and the known connections of Hxk1 to carbon metabolism.

8) The accompanying Chen et al. paper provides evidence that Ndt80 is required for induction of the short *NDC80^ORF^* mRNA. If so, how can it be induced in the experiment of Figure 5A conducted with an ndt80 mutant?

9) Figure 5. Panel B lacks an RNA loading control and quantitation. The data in panel E need to be normalized to total H3 levels.

10) Figure 6B needs to be accompanied by measurements of Ndc80 protein to confirm that the requirement for Set2/Set3 in Ndc80p repression have been bypassed. Similarly for Figure 6C.

11) Figure 7. The figure does not add very much to the discussion. The vertical red lines are not defined. The nucleosomes look more like Pol II molecules with modified CTDs. Please modify accordingly.

12) In the Discussion, the authors conclude that their data show the "co-transcriptional recruitment of Set1/Set3C and Set2/Rpd3S is essential for establishing a repressive chromatin state and inhibiting *NDC80^ORF^* transcription". Recruitment of none of these factors was tested and therefore the text needs to be modified accordingly.

*Non-essential revisions:*

1) Figure 2 provides the main data supporting a transcriptional interference mechanism. As presented, the data are insufficient to support the stated conclusions. First, additional factors should be assayed by ChIP, beyond Sua7. In particular, how does *NDC80^luti^* expression influence Ndt80 occupancy in the *NDC80* promoter region? Second, what is the effect of blocking *NDC80^luti^* transcription on Sua7 and Ndt80 occupancy? What is the effect of blocking *NDC80^luti^* on nucleosome occupancy? Note: the additional ChIP experiments suggested in this point are encouraged, but are not considered essential for publication. However, if the authors choose not to strengthen this part of the study, it is essential that they substantially modulate the strength of their statements in the text to be in line with what the data actually demonstrate.

2) Figure 4. The authors use set2∆ as a proxy for removing Rpd3S. The HDAC could be directly tested by performing similar northern and western blots with an rco1∆ strain. The authors argue that loss of the two HDAC systems alleviates transcriptional interference. This could be tested by ChIP of Sua7 in the HDAC mutant strains. Surprisingly, the authors show effects of deleting SET2 and SET3 on nucleosome occupancy but not histone acetylation levels at the ORF promoter. Their model should be tested more directly with better choices of mutants and ChIPs. Note: the additional ChIP experiments suggested in this point are encouraged, but are not considered essential for publication. However, if the authors choose not to strengthen this part of the study, they need to substantially modulate the strength of their statements in the text to be in line with what the data actually demonstrate. For example, without directly testing recruitment of HDACs, which can be tricky to do, they cannot say that their data show recruitment of HDACs via H3K36me3 and H3K4me2. They can merely describe this as a likely scenario. However, they could provide more support for this model by measuring histone acetylation levels, which is not hard to do.

3) Figure 1 essentially recapitulates the same or similar experiments described in the accompanying paper by Chen et al., with somewhat different promoter replacements or termination insertions for the *NDC80^luti^* transcript. Given this redundancy, the authors should consider omitting this figure. If they prefer to retain the figure, substantial improvements are needed. Here, the corresponding Ndc80 protein data are lacking for these promoter and termination constructs; although the quality of the northern data is better here for the terminator construct. However, mRNA loading controls and quantification of the northern data from replicates is lacking and should be provided.

4) It's surprising in Figure 2C that the nucleosome peak at ~-500, which is probably the +1 nucleosome for the *luti* mRNA promoter doesn't decline in prophase on induction of this transcript. This deserves some comment.

5) Figure 2B: It's surprising that TFIIB binding increased the most during prophase at -800, >300 bp upstream of the TSS for the *luti* transcript, raising questions about the location of the *luti* promoter. This deserves some comment.

6) Integrate Figure 3—figure supplement 1 data into Figure 3, and add premeiotic and S-prophase labels to data key.

7) Subsection “Gene repression by *NDC80^luti^* transcription is tunable”, last paragraph: "repression" should probably be "expression".

8) Subsection “*NDC80^luti^* mediated repression of *NDC80^ORF^* is dynamic”, end of first paragraph: it would be helpful to cite explicitly the data that supports this statement.

9) It would be nice to see a northern analysis to examine both long and short *NDC80* mRNAs in Figure 6A. This would provide support for the last two sentences in the first paragraph of the subsection “Gene repression by *NDC80^luti^* transcription is tunable”.

# Reply: Author response

**DOI:** 10.7554/eLife.27420.016

Essential revisions:

1) The increase in MNase-resistance in the NDC80^ORF^ promoter region is not necessarily due to nucleosomes as it has been shown that non-histone protein binding in NDRs can also confer MNase-resistance (PMID: 28157509). For this reason, a ChIP of the MNase-resistant chromatin with histone antibodies is required to establish nucleosome versus non-histone protein occupancy.

We now performed the experiment with histone H3 ChIP on MNase treated chromatin extracts. The results are similar to our original observation. We concluded that nucleosomes bind the NDR region of the *NDC80* locus when *NDC80^luti^* is transcribed.

2) In Figure 2C changes in nucleosome occupancy are inferred upon repression of the downstream promoter (at -100), but there is no evidence for loss of nucleosomes accompanying activation of the upstream promoter. Is this because the upstream promoter is nucleosome free even when repressed or is there another explanation?

We have two potential explanations:

1) The transcriptional repressor of *NDC80^luti^*, Ume6, binds to the upstream promoter (see Chen et al), which could exclude nucleosomes from binding to the same sequence. When *NDC80^luti^* is activated by Ime1-Ume6, the region remains relatively unchanged, because Ume6 binding to the promoter occurs prior to Ime1-dependent activation. Hence, the upstream promoter remains free of nucleosomes prior and during activation of *NDC80^luti^* transcription.

2) Alternatively, the Ume6 repressor keeps the +1 nucleosome in place. When Ime1 is expressed together with Ume6, it activates *NDC80^luti^* and its transcription causes the +1 nucleosome to remain positioned.

3) Figure 2. Some information on the sequences of the promoters should be presented. Does the Sua7 occupancy profile make sense with respect to positions of core promoter elements? Why is the highest Sua7 signal detected 400bp upstream of the NDC80^luti^ TSS?

We have attempted to look for core promoter elements, which in yeast are not so well characterized, except for the TATA element. We were not able to identify an obvious TATA element in the *NDC80* promoter, which is consistent with its previous classification as a TATA-less promoter (PMID: 15006352). Sua7 binding is approximately at -100 bp from AUG, which is within the region where core promoter elements are expected in yeast (PMID: 15006352).

The Sua7 signal in the upstream promoter region can be explained by expression of the neighbouring gene, *PAN6*, which is transcribed in the opposite direction compared to *NDC80*. We observed that *PAN6* expression increases during entry into meiosis, which may explain the reason for the broad peak of Sua7 binding.

4) Figure 2. How were the nucleosome positioning data normalized? Typically these data are normalized to a known, highly occupied nucleosome position (for example, Sekinger et al. Mol Cell 18: 735). Also control gels should be included to show the MNase digestion profile.

We have now included examples of how we prepared the MNase treated extracts, and show the MNase digestion pattern on a gel (Figure 1—figure supplement 1E and Figure 3—figure supplement 1G). We have performed ChIP with histone H3 on extracts with predominantly mononucleosomal DNA fragments. We have normalized our data to *PHO5* sequences. This control gave consistent results.

5) ChIP with histone antibodies is also required to normalize the H3-K36me3 and H3-K4me2 ChIP signals in Figure 3A-D and Figure 3—figure supplement 1 in order to establish that the amount of methylation per nucleosome is increased over the NDC80^ORF^ promoter in a manner dependent on the luti promoter. K4me2 levels should be included in Figures 3C and 3D.

We have now performed histone H3 ChIPs for Figure 2 (old Figure 3) and used this to normalize our H3K4me2 or H3K36me3 signals. We have also included K4me2 ChIPs in a mutant that does not express NDC80_luti (*ndc80^-600-30^*) in premeiotic cells and S-prophase cells (Figure 2C). It is worth noting that in the old version we used a different mutant for the K36me3 experiment (*ndc80^-600-500^*). To keep it consistent in the revised manuscript, we repeated the experiment with the *ndc80^-600-300^*mutant (Figure 2D). The results are comparable between the two mutants. Finally, we checked K4me2 by ChIP when *NDC80^luti^* is expressed from the *GAL* promoter in Figure 2E.

6) Figure 4A: RNA loading controls are needed in the northern blots. Quantification of the mRNA from replicates with a loading control is needed to establish that the NCD80^ORF^ transcript is elevated reduced in prophase in the double mutant relative to WT, as this seems to be limited to the 3hr timepoint, and even this increase is not evident in Figure 4D. Perhaps the only meaningful increase in terms of Ndc80p expression occurs at 4-5hr.

We have now performed loading controls for the northern blots in Figure 3A and quantified the experiment. Different time-course experiments (biological replicates) always give the same trends, but do not always show the same level of changes. One explanation is that time course experiments have inherent variability with regards to meiotic kinetics between biological replicates. This makes it tricky to take the average of multiple experiments, because the variation in mRNA level is due to both the biological variation as well as the variation in meiotic synchrony. Furthermore, in experiments where the wild type and mutant samples were run on different gels, they were transferred to different blots and hybridized separately, which could also cause technical variability.

Therefore, we only show representative experiments for Figure 3A and 3D (old Figure 4A and 4D). To address this issue, we further examined the *NDC80^orf^* levels of selective time-points from three independent experiments in Figure 3E, for the earlier meiotic time points, where the *pCUP-IME1/IME4* system yields better synchrony. For each experiment, the wildtype and mutant samples were run on the same gel/blot/hybridization. We observed significant differences in *NDC80^orf^*expression at 3.5 and 4.5 hours between the wild-type and *set2 set3* double mutant, but not the single mutants.

Please note that we replaced the experiment in Figure 3D with another repeat because hybridization with the *SCR1* loading failed.

7) Figure 4G. How many repetitions were done to calculate the relative Ndc80 levels? Also, the levels of the Hxk1 loading control seem to be changing both in this panel and in Figure 4—figure supplement 1. This is perhaps not surprising given the change in carbon source in this experiment and the known connections of Hxk1 to carbon metabolism.

The Figure 3H (formerly Figure 4G) shows the quantification for that particular repeat. We have now included the quantification from two independent experiments in Figure 3—figure supplement 1E. There was no change in carbon source in this experiment. The samples were treated with β-estradiol that induces the *GAL* promoter using Gal4-ER fusion.

8) The accompanying Chen et al. paper provides evidence that Ndt80 is required for induction of the short NDC80^ORF^ mRNA. If so, how can it be induced in the experiment of Figure 5A conducted with an ndt80 mutant?

Our data suggest that there at least two ways to activate *NDC80^orf^* transcription. One is during regular mitotic cell cycle of which we do not know the corresponding transcription factors. This is also seen from the fact that *NDC80^orf^*is expressed before entry into meiosis. The second way is during meiosis by the transcription factor Ndt80. In Figure 4 (old Figure 5), we examined how *NDC80^orf^* transcription is reactivated when cells are switched from meiotic entry back to mitotic growth. We propose that the transcription factors important for *NDC80^orf^* expression in mitosis controls the re-activation during return to mitotic cell cycle. The accompanying paper by Chen et al. proposes that Ndt80 bypasses *NDC80^luti^* mediated repression of *NDC80^orf^*. Therefore we performed the experiment in an *ndt80* mutant background, the induction of *NDC80^orf^*in an *ndt80* mutant is consistent with mitotic mediated activation of *NDC80^orf^*.

9) Figure 5. Panel B lacks an RNA loading control and quantitation. The data in panel E need to be normalized to total H3 levels.

We have now included a loading control and normalized the ChIP signals to histone H3. We have quantified the levels of both *NDC80^orf^* and *NDC80^luti^* from two independent experiments in Figure 4C.

10) Figure 6B needs to be accompanied by measurements of Ndc80 protein to confirm that the requirement for Set2/Set3 in Ndc80p repression have been bypassed. Similarly for Figure 6C.

We now included Ndc80 protein levels for Figure 5B and 5C (old Figure 6B and 6C) and in Figure 5—figure supplement 1B-E. Example western blots and the mean of at least two independent experiments are shown in these figures.

11) Figure 7. The figure does not add very much to the discussion. The vertical red lines are not defined. The nucleosomes look more like Pol II molecules with modified CTDs. Please modify accordingly.

We have now changed the figure, accordingly.

12) In the Discussion, the authors conclude that their data show the "co-transcriptional recruitment of Set1/Set3C and Set2/Rpd3S is essential for establishing a repressive chromatin state and inhibiting NDC80^ORF^ transcription". Recruitment of none of these factors was tested and therefore the text needs to be modified accordingly.

We have removed the terms Set1/Set3C and Set2/Rpd3S from most parts of the manuscript, and mention Set2 and Set3 only. We discuss Set1/Set3C and Set2/Rpd3S only in the Discussion and Introduction.

Non-essential revisions:

1) Figure 2 provides the main data supporting a transcriptional interference mechanism. As presented, the data are insufficient to support the stated conclusions. First, additional factors should be assayed by ChIP, beyond Sua7. In particular, how does NDC80^luti^ expression influence Ndt80 occupancy in the NDC80 promoter region? Second, what is the effect of blocking NDC80^luti^ transcription on Sua7 and Ndt80 occupancy? What is the effect of blocking NDC80^luti^ on nucleosome occupancy? Note: the additional ChIP experiments suggested in this point are encouraged, but are not considered essential for publication. However, if the authors choose not to strengthen this part of the study, it is essential that they substantially modulate the strength of their statements in the text to be in line with what the data actually demonstrate.

We agree that ChIP for more factors is desirable. We have tried to ChIP for several factors at the *NDC80^orf^* promoter. We tested sequence specific transcription factors that have been implicated in regulating *NDC80* expression by genome-wide studies: Hcm1, Swi4, and Fkh1. In addition, we have tried to ChIP for basal transcription factors such as: Taf1 and TBP. Unfortunately, none the factors gave a reproducible ChIP signal. One explanation is that *NDC80^orf^* expression quite low during mitosis (only a few mRNA copies per cell). This makes it is challenging to obtain robust ChIP signals. We have modulated our statements and only talk about TFIIB (as this is only factor that worked) and not preinitiation complex.

2) Figure 4. The authors use set2∆ as a proxy for removing Rpd3S. The HDAC could be directly tested by performing similar northern and western blots with an rco1∆ strain. The authors argue that loss of the two HDAC systems alleviates transcriptional interference. This could be tested by ChIP of Sua7 in the HDAC mutant strains. Surprisingly, the authors show effects of deleting SET2 and SET3 on nucleosome occupancy but not histone acetylation levels at the ORF promoter. Their model should be tested more directly with better choices of mutants and ChIPs. Note: the additional ChIP experiments suggested in this point are encouraged, but are not considered essential for publication. However, if the authors choose not to strengthen this part of the study, they need to substantially modulate the strength of their statements in the text to be in line with what the data actually demonstrate. For example, without directly testing recruitment of HDACs, which can be tricky to do, they cannot say that their data show recruitment of HDACs via H3K36me3 and H3K4me2. They can merely describe this as a likely scenario. However, they could provide more support for this model by measuring histone acetylation levels, which is not hard to do.

We agree that our model predicts an increase in histone acetylation. However, given that we had put a lot of effort and time in completing the other essential and non-essential comments, we decided to test this further in a follow up study. We have modulated our conclusions in the Results section and refer the Set2 and/or Set3. We only refer to the Set1/Set3C and Set2/Rpd3S pathways in the Discussion.

3) Figure 1 essentially recapitulates the same or similar experiments described in the accompanying paper by Chen et al., with somewhat different promoter replacements or termination insertions for the NDC80^luti^ transcript. Given this redundancy, the authors should consider omitting this figure. If they prefer to retain the figure, substantial improvements are needed. Here, the corresponding Ndc80 protein data are lacking for these promoter and termination constructs; although the quality of the northern data is better here for the terminator construct. However, mRNA loading controls and quantification of the northern data from replicates is lacking and should be provided.

We have removed Figure 1 from the manuscript, and made a new Figure 1 consisting of parts of the old Figure 1 and the complete Figure 2. We included the first data panel of the old Figure 1 in the new Figure 1, for which we added a loading control and quantified the signals from the northern and western blot.

4) It's surprising in Figure 2C that the nucleosome peak at ~-500, which is probably the +1 nucleosome for the luti mRNA promoter doesn't decline in prophase on induction of this transcript. This deserves some comment.

We agree that it is somewhat surprising that there was no shift in nucleosome occupancy around that +1 positon. Given that this a PCR based assay, we cannot exclude that there are subtle changes that we missed. Given that Ume6 binds the region prior to its activation, perhaps the Ume6 repressor keeps the +1 nucleosome in place. When Ime1 is expressed together with Ume6, it activates *NDC80^luti^* and transcription causes the +1 nucleosome to remain positioned.

5) Figure 2B: It's surprising that TFIIB binding increased the most during prophase at -800, >300 bp upstream of the TSS for the luti transcript, raising questions about the location of the luti promoter. This deserves some comment.

*PAN6* transcribed in the divergent direction is directly adjacent *NDC80^luti^*. Its expression increases upon meiotic entry, which may explain the shift in peak. See also point 3 of Essential revisions.

6) Integrate Figure 3—figure supplement 1 data into Figure 3, and add premeiotic and S-prophase labels to data key.

Has been added, accordingly.

7) Subsection “Gene repression by NDC80^luti^ transcription is tunable”, last paragraph: "repression" should probably be "expression".

Has been changed, accordingly.

8) Subsection “NDC80^luti^ mediated repression of NDC80^ORF^ is dynamic”, end of first paragraph: it would be helpful to cite explicitly the data that supports this statement.

We now changed the section:

“Interestingly, the MSE site (approximately -200 bp upstream from the AUG) in the *NDC80^ORF^* promoter is not protected by nucleosomes even during *NDC80^luti^* transcription (Figure 1E), which may explain the ability of Ndt80 to activate *NDC80^ORF^* while NDC80^luti^ is expressed.

The dynamic nature of the regulation is also illustrated by our finding that *NDC80^luti^* mediated repression can be rapidly reversed (Figure 4).”

9) It would be nice to see a northern analysis to examine both long and short NDC80 mRNAs in Figure 6A. This would provide support for the last two sentences in the first paragraph of the subsection “Gene repression by NDC80^luti^ transcription is tunable”.

We agree that this is would further support our data. However, the experiment is not trivial. The northern blot can be tricky to quantify because the background signal around the relatively weak *NDC80^orf^* mRNA band will most likely increase when *NDC80^luti^* mRNA is highly expressed, owing to the close proximity of the transcripts. Therefore we have measured Ndc80 protein levels in cells harbouring no or 8 copies of *lexO* sites in the *NDC80^luti^* promoter instead. We used different concentrations of β-estradiol to measure the effect on Ndc80 protein levels. The differences in growth inhibition observed in the spot assay were also reflected at the Ndc80 protein level (see Figure 5—figure supplement 1B and C).
